# Potential of Dietary Agent Daidzein in Cancer Prevention and Treatment: Opportunities and Challenges

**DOI:** 10.3390/cancers18101639

**Published:** 2026-05-19

**Authors:** Subhadas Chatterjee, Sabyasachi Banerjee, Sankhadip Bose, Kumar Ganesan, Cassandra R. Reilly, Anupam Bishayee

**Affiliations:** 1 Department of Pharmacology, Bengal School of Technology, Chuchura 712 102, West Bengal, India; 2 Department of Pharmaceutical Chemistry, Gupta College of Technological Sciences, Asansol 713 301, West Bengal, India; 3 School of Pharmacy, The Neotia University, Sarisa 743 368, West Bengal, India; 4 School of Chinese Medicine, Li Ka Shing Faculty of Medicine, The University of Hong Kong, Hong Kong 999077, China; 5 Department of Pharmacology, College of Osteopathic Medicine, Lake Erie College of Osteopathic Medicine, Bradenton, FL 34211, USA

**Keywords:** daidzein, anticancer, apoptosis, antiproliferation, mechanistic pathways, systematic review

## Abstract

**Background:** Daidzein, a secondary metabolite primarily obtained from soybean (*Glycine max* L.) and other legumes, has significant nutritional and pharmacological value. Chemically, daidzein is an isoflavone and plays a crucial role in the therapeutic amelioration of numerous disorders, including allergies, inflammation, diabetes, cardiovascular, and neurodegenerative diseases. Emerging preclinical evidence suggests potential antineoplastic activity of daidzein against various cancers. This current work aims to perform a critical evaluation of daidzein’s potential as an anticancer molecule with an in-depth understanding of its mechanisms of action. **Methods:** The data for this review were obtained from various sources, including PubMed, Scopus, and Web of Science. **Results:** Daidzein, as a pure phytochemical or in combination with other phytochemicals and anticancer drugs, has been reported to induce apoptotic and autophagic cell death, impeding cell growth, viability, proliferation, and angiogenesis, and arresting cell division at various phases in vitro. Various daidzein formulations also exhibited similar anticancer effects by immunomodulation and genetic alteration in the cancer cells. In vivo anticancer studies of daidzein also suggest modulation of several hallmark pathways, such as inhibition of nuclear factor-κB, Janus kinase/signal transducer and activator of transcription, and rat sarcoma virus/rapidly accelerated fibrosarcoma. **Conclusions:** Irrespective of numerous promising preclinical studies, the absence of clinical studies provides a major challenge to establishing daidzein’s safety and efficacy in human cancers. Therefore, further advancements in clinical research of daidzein are vital for manifesting as an antineoplastic drug.

## Simple Summary

Cancer is one of the leading causes of death across the world today. As a result, many scientists and researchers are interested in the ability of natural compounds to help prevent or treat cancers. Daidzein is a compound that is naturally derived from soybeans and legumes, which are consumed by many individuals across the world. Experimental research studies show that daidzein may exhibit anticancer properties, which suggest that daidzein may help to prevent or treat cancers. Thus, the purpose of this work is to evaluate research studies regarding the anticancer properties of daidzein and related mechanisms of action. Through gaining an understanding of such mechanisms of action, researchers may be able to better direct future research efforts into the potential of dietary agent daidzein in the prevention or treatment of cancer.

## 1. Introduction

Cancer is one of the leading causes of death worldwide. According to the most recent estimates from Global Cancer Observatory (GLOBOCAN) for 2022, there were 20 million new cases of cancer diagnosed worldwide, along with 9.7 million cancer deaths from all types of cancer, including non-melanoma skin cancers [1]. Projections of the number of new cases of cancer each year suggest that the number will rise to 35 million by 2050. Lung cancer was the most commonly diagnosed form of cancer, with 2.5 million new cases accounting for 12.4% of all cancer diagnoses, and was also the leading cause of cancer-related deaths, with 1.8 million cancer deaths from lung cancer. Breast, colorectal, prostate, and stomach cancers were the other three most commonly diagnosed types of cancer. These statistics illustrate the need for effective strategies both for the prevention of and the treatment of cancer patients, especially those with lung cancer, and those with high levels of toxicity associated with their treatments [1].

In the modern world, the use of phytochemicals for the treatment of various diseases, specifically cancer, has gained new insight in the last few decades [2]. The use of phytochemicals for developing new drugs is much favored for their inherent bioactivity and facilitated cellular uptake. Diet plays a major role in chemoprevention, as various phytochemicals, either as a pure compound or in combination with other major chemotherapeutic agents, ameliorate cancer progression at various stages [3]. Several notable modifications, such as scavenging of reactive oxygen species (ROS) formed due to improper metabolism [4], regulating apoptotic and autophagic pathways, or diminishing cell proliferation, migration, and cell cycle [5,6,7,8]. Plant secondary metabolites are mainly classified into five types, specifically alkaloids, glycosides, phenolics, terpenoids, and sulfur-containing compounds, which possess significant importance in cancer chemotherapy. Among the large class of phytochemicals, isoflavones have gained special attention regarding their extensive therapeutic advantages as they mimic the structure and function of endogenous estrogens [9].

**Figure 1:**
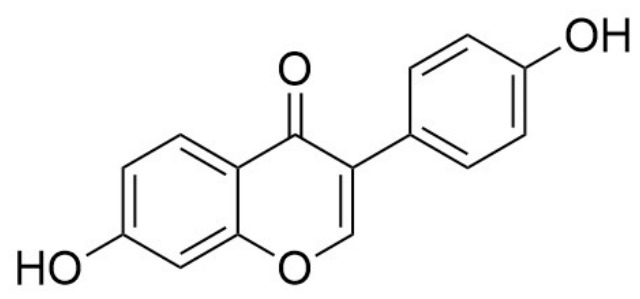
Chemical structure of daidzein.

Daidzein (Figure 1), which is chemically an isoflavone and functionally a phytoestrogen, has gained new therapeutic insight for its multifaceted pharmacological actions. Daidzein is primarily found in many leguminous plants, such as soybeans (*Glycine max*), red clover (*Trifolium pratense*), and alfalfa (*Medicago sativa*) [10,11]. Due to the rich nutraceutical importance of daidzein, it has been used in soy flour, soy protein isolates, miso, tempeh, tofu, and soy-based infant formulas. Despite its presence in various foods, the concentration of daidzein present in these sources varies significantly, ranging from 22 mg in a half cup of miso to 7 mg in one cup of soy milk. As daidzein is converted into its active metabolite in the gut, most of the pharmacological effects of this phytocompound resemble those of 17β-estradiol, and hence can exert both estrogenic and antiestrogenic effects in the body [12,13].

Beyond possessing prominent endocrine activity in the body, daidzein also exhibits significant potential for curing various ailments, independent of its modulation of the endocrine pathways [14]. This phytoestrogen exerts anti-inflammatory, anti-asthmatic, anti-allergic, and antimicrobial effects. Furthermore, it provides significant organ-specific protection, including hepatoprotective, nephroprotective, and neuroprotective properties [10,11,15,16,17,18]. These benefits are mediated through modulation of key signaling pathways, specifically by mitigating oxidative stress and downregulating the expression of pro-inflammatory cytokines, such as interleukin (IL)-1β and tumor necrosis factor-α (TNF-α) [12,19]. Moreover, regulation of nuclear factor-κB (NF-κB), mitogen-activated protein kinase (MAPK), and nuclear factor erythroid 2-related factor 2 (Nrf2) signaling pathways also play a vital role in daidzein in exhibiting such pharmacological effects [20,21,22,23,24,25,26].

**Figure 2:**
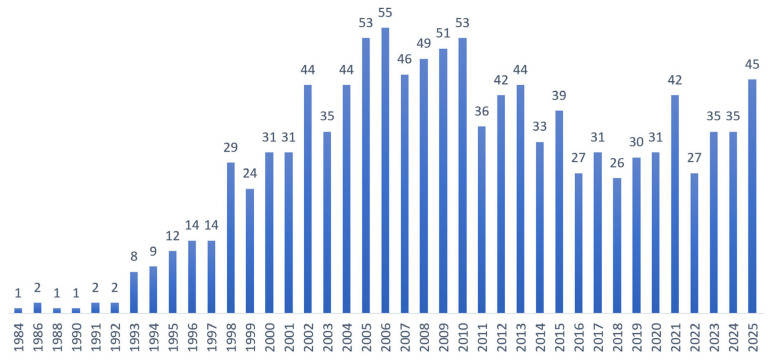
Consistent escalation in the publication numbers based on search using keywords “daidzein” and “cancer” in PubMed database.

In the last few decades, daidzein has become a subject of growing interest as a potential anticancer agent. A steady rise in peer-reviewed publications on “daidzein and cancer” has been observed since 2002 (Figure 2). Previous reviews have addressed aspects of daidzein’s pharmacology or the broader role of soy isoflavones in cancer [27,28,29,30,31,32,33]. However, most were narrative in scope, focused on soy isoflavone mixtures rather than isolated daidzein, or were restricted to a single cancer type [34]. A recent narrative review by Islam et al. [35] provided a useful overview of daidzein’s anticancer activity across various cancers and highlighted selected mechanistic pathways. Nevertheless, that work remained descriptive, offered no formal risk-of-bias assessment, did not systematically evaluate daidzein in combination with other agents or in nanoformulations, and provided limited critical appraisal of translational limitations such as dosing relevance, selectivity, and biphasic effects.

The present review addresses these gaps by providing an up-to-date, narrative synthesis with systematic literature search elements that critically evaluates the preclinical anticancer effects of pure daidzein, daidzein in combination therapies, and daidzein-based nanoformulations across multiple cancer types. By incorporating a risk-of-bias assessment for in vivo studies, a quantitative overview of the evidence base, and an explicit discussion of methodological limitations such as model heterogeneity, concentration relevance, potential biphasic/estrogenic effects, and the absence of clinical data on pure daidzein, this work clarifies the current strength of evidence and identifies specific knowledge gaps that must be filled before meaningful clinical translation can occur. Such a comprehensive evaluation is timely because daidzein’s complex phytoestrogenic properties may produce both beneficial and potentially adverse outcomes in hormone-sensitive cancers, underscoring the need for a balanced, critical assessment across diverse models.

## 2. Methodology for Literature Search and Study Selection

The current review was performed in accordance with the Preferred Reporting Items for Systematic Reviews and Meta-Analyses (PRISMA) 2020 guidelines [36,37]. This review has not been registered with any database, such as International Prospective Register of Systematic Reviews (also known as PROSPERO). All literature databases were last searched on 31 March 2026, and no database-specific date restrictions were applied prior to this cut-off, to identify experimental studies exploring the anticancer activities of daidzein as a pure phytochemical as well as in combination with other chemotherapeutic agents and various nanoformulations in relation to in vitro and in vivo research data and molecular mechanisms. Clinical studies for the same were searched using ClinicalTrials.gov.

### 2.1. Databases and Keywords

A comprehensive search of the prominent research databases, specifically PubMed, Scopus, Web of Science, Cochrane Library, and ClinicalTrials.gov, was conducted to find relevant literature. Within each of the databases, searches were performed using exact search strings for all articles, irrespective of language or date of publication (except for the most recent date of publication for each database). The PubMed database was searched using the search string (“daidzein”[MeSH Terms] OR “daidzein”[All Fields] OR “daidzin”[All Fields] OR “isoflavone”[MeSH Terms] OR “phytoestrogen”[All Fields]) AND (“neoplasms”[MeSH Terms] OR “cancer”[All Fields] OR “anticancer”[All Fields] OR “antineoplastic”[All Fields] OR “carcinoma”[All Fields] OR “tumor”[All Fields] OR “tumour”[All Fields]) AND (“in vitro”[All Fields] OR “cell line”[All Fields] OR “in vivo”[All Fields] OR “animal model”[All Fields] OR “xenograft”[All Fields] OR “clinical”[All Fields] OR “mechanism”[All Fields] OR “signaling pathway”[All Fields]). The Scopus database was searched using TITLE-ABS-KEY (daidzein OR daidzin OR isoflavone OR phytoestrogen) AND TITLE-ABS-KEY (cancer OR neoplasm OR carcinoma OR tumor OR tumour OR anticancer OR antineoplastic) AND (TITLE-ABS-KEY (“in vitro” OR “cell line” OR “in vivo” OR xenograft OR “animal model” OR mechanism OR “signaling pathway”)). The Web of Science database was searched using TS = (daidzein OR daidzin OR isoflavone OR phytoestrogen) AND TS = (cancer OR neoplasm OR carcinoma OR tumor OR tumour OR anticancer) AND TS = (“in vitro” OR “cell line” OR “in vivo” OR xenograft OR mechanism OR “signaling pathway”). The Cochrane Library database was searched using (“daidzein” OR “daidzin” OR isoflavone OR phytoestrogen) AND (cancer OR neoplasm OR carcinoma) in Title, Abstract, Keyword. The ClinicalTrials.gov registry was searched using (daidzein OR daidzin) AND (cancer OR neoplasm OR carcinoma OR tumor).

All the records retrieved through the search (*n* = 119 after initial deduplication) were imported into EndNote 21. Automatic detection of duplicates was performed using the “Find Duplicates” function for each of the records (using fields for author, year, title, and DOI). Suspected duplicates were manually removed by two independent reviewers, resulting in 92 unique records to be screened for title and abstract relevance.

**Figure 3:**
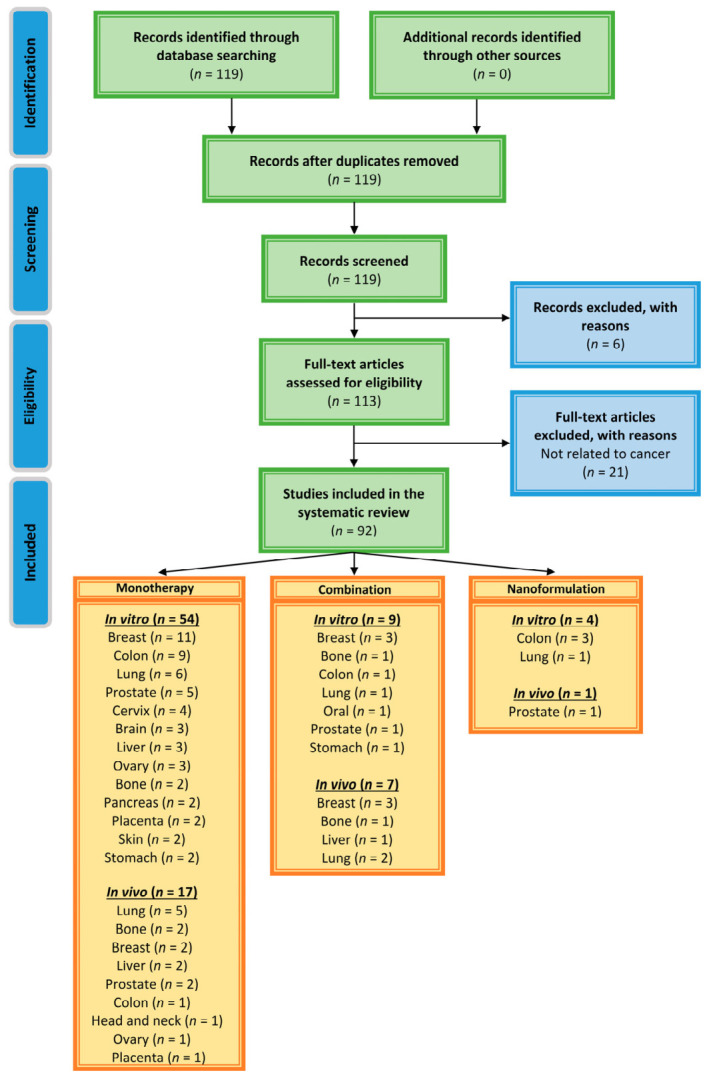
PRISMA flow diagram of the literature search and study selection process. A total of 119 records (publications) were identified through database searching and other sources. After screening the titles and abstracts of these publications, 67 publications were included in the review. However, some of these publications included more than one independent experimental dataset. Thus, 92 individual studies and datasets were analyzed in this review. The study selection process depicted in the diagram is at the level of the publication; the analyses performed in this review are at the level of individual experimental datasets.

Title and abstract screening were performed independently by two reviewers. Full-text assessment was conducted independently by all authors. Any disagreements were resolved by consensus discussion involving a third senior reviewer. Reasons for exclusion at each stage are detailed in the PRISMA flow diagram (Figure 3). 

### 2.2. Inclusion Criteria

The inclusion criteria used for selecting the relevant articles were established through a framework called Participants, Intervention, Comparator, and Outcomes (PICO). The ‘participants’ included human cancer cell lines or animal tumor models. The ‘intervention’ comprised pure daidzein (as a single phytochemical), daidzein in combination with other agents, or daidzein-based nanoformulations. The ‘comparator’ consisted of vehicle-treated controls or, where available, standard chemotherapeutic agents. The ‘outcomes’ included any measure of anticancer effect, including cell viability, proliferation, apoptosis, cell cycle arrest, migration/invasion, tumor growth, or modulation of cancer-related signaling pathways.

Only full-text articles that fulfilled the following criteria were selected for a thorough analysis: (i) research articles that were originally published within scientific journals, (ii) articles that were written in the English language, (iii) articles that focused on the anticancer effects of daidzein as a pure phytocompound as well as in combination with other chemotherapeutic agents and various nanoformulations, and (iv) articles that were related to experiments that used cancer cell lines or animal tumor models. The search was limited to English-language publications to ensure consistency, accuracy, and ease of interpretation of the included studies. The main disadvantage of utilizing only English-language publications is that studies published in other languages may have been excluded from this review. Such language bias potentially excludes studies from regions of the world that may be publishing research on soy products and isoflavones in particular. However, consideration of this potential bias has been incorporated into the discussion and interpretation of the findings from the review.

### 2.3. Exclusion Criteria

The studies were excluded based on any of the criteria listed here: (i) review articles, book chapters, systematic reviews, meta-analyses, editorials, abstracts from conferences, and unpublished statistics, (ii) studies not conducted on cancer cell lines and/or animal tumor models, (iii) studies not published in English, (iv) studies unrelated to cancers that describe the biological or pharmacologic properties of daidzein, and (v) studies that were retracted and/or methodologically flawed. The studies that were excluded from this review were those that were systematic reviews and meta-analyses in order to avoid potential duplication of data and potential bias from secondary analyses of the studies in question.

### 2.4. Data Synthesis

Initially, a total of 119 articles related to the anticancer properties of daidzein were obtained from the selected databases. After the screening process, a total of 67 articles, comprising in vitro, in vivo combination, and nanoformulation-based studies, were left for thorough qualitative analysis. These 67 publications yielded 92 distinct studies/datasets because several papers reported multiple independent experiments. Importantly, some publications included more than one independent experimental dataset (e.g., different cell lines, in vitro and in vivo experiments, or different treatment conditions), each of which was analyzed separately. Thus, a total of 92 individual studies and datasets were included for analysis. While the PRISMA flow diagram reports the number of publications that meet the inclusion criteria, the analyses and results presented in the manuscript report the number of independent experimental datasets that were performed within those publications. In order to ensure the transparency, reproducibility, and organization of the diverse preclinical study data, a strategy was developed to process the data. From each of the included studies, data to be gathered and processed will include information regarding the type of model that was used (cell lines or animal models), the doses that were used, the length of time that the treatment was applied, the type of treatment that was applied (pure daidzein, combination treatments, or nanoformulations of daidzein), and the outcomes and mechanisms relating to the anticancer properties of daidzein as reported in each study. Due to the diversity of treatments and outcomes among the included preclinical studies, the extraction and organization of such data is essential to enable any comparisons to be made between the studies. Accordingly, such a strategy ensures the study adheres to the recommendations of the PRISMA statement regarding systematic reviews of scientific studies. No publication year filtering was used in selecting the articles. The last literature search was executed in March 2026. The ClinicalTrials.gov database revealed that there are no available studies focusing on the use of daidzein in pure form, as well as in combinations or nanoformulations, as an anticancer agent. Because of the heterogeneity among the studies, it was not possible to perform a meta-analysis on these studies, and thus, the results should be considered with caution when comparing the findings from different studies. Two reviewers independently extracted data using the above framework, and any disagreements were resolved through discussion. The distinction between publications and individual datasets is consistent with evidence synthesis practices in preclinical research, where a single article may contribute multiple independent experimental units for analysis.

### 2.5. Review Process

The selection of studies was done through a critical and independent evaluation undertaken by all the authors, in a manner that satisfies the predetermined criteria of inclusion and exclusion. The screening of titles and abstracts and the assessment of full texts were performed by at least two reviewers independently. Inter-reviewer agreement was assessed qualitatively during the screening of the studies to ensure that in cases in which the two reviewers had different judgments regarding the eligibility of a particular study, the two reviewers could reach a consensus regarding the eligibility of that study. In cases in which the reviewers were unable to reach a consensus, a third and senior reviewer was consulted. In addition to searching the databases for articles that relate to the topic of review, the reference lists of relevant review articles and research papers were manually screened to ensure that no additional studies were published during the period in which they were published. These additional studies were also screened according to their relevance to the anticancer effects of daidzein.

### 2.6. Risk of Bias Within Studies

For the in vivo animal studies, the quality of methodology was evaluated by the Systematic Review Center for Laboratory Animal Experimentation’s (SYRCLE) risk-of-bias (RoB) assessment, which was a Cochrane RoB assessment tailored to animal intervention studies [38]. The SYRCLE RoB assessment contains a checklist of domains of possible bias, including bias in the selection of studies, performance bias, bias in the detection of outcomes, bias due to attrition, reporting bias, and other sources of bias. A total of 18 in vivo studies, examining the anticancer effect of daidzein, were identified to be examined for risk of bias, following the SYRCLE’s RoB assessment guidelines. These 18 studies [39,40,41,42,43,44,45,46,47,48,49,50,51,52,53,54,55,56] represent all of the in vivo animal experiments that were identified through the search, even though several of the publications also included in vitro experiments. However, in these cases, only the in vivo experiments with the pure compound were considered for assessment with the SYRCLE tool. The risk-of-bias graph and the corresponding risk-of-bias summary were generated using Review Manager (RevMan) software, version 5.4.1 (Cochrane Collaboration, London, UK). Since no tool has yet been established as being both accepted and standardized to assess the quality of in vitro studies examining the anticancer properties of phytochemicals, no assessment of the risk of bias for these types of studies was performed; however, the limitations of these studies were considered during the interpretation of their data.

## 3. Therapeutic Activities of Daidzein Against Cancer

In preclinical studies, daidzein has been shown to be an interesting naturally occurring isoflavone with reported anticancer-related biological activities. Numerous in vitro and in vivo studies have shown that daidzein can impact the proliferation and apoptosis of cancer cells. However, most of these studies are based on preclinical models of cancer. Thus, these studies have provided insights into the biological activities of daidzein.

### 3.1. Brain Cancer

One of the rarest forms of cancer, brain cancer ranks 19th among incidence rates globally and causes 248,305 deaths annually. Due to the complex histology, brain cancer can be classified into more than 100 types, with glioma identified as the most common [57]. Glioblastoma is a frequent and aggressive form of glioma, emerging spontaneously from the glial cells and may develop into lower-grade or anaplastic astrocytoma [58]. It is characterized by overexpression of molecular markers such as methylation of O^6^-methylguanine DNA methyltransferase promoter and mutation of isocitrate dehydrogenase [59,60].

In a study by Siegelin et al. [61], daidzein was evaluated for its anticancer effect against LN229 (p53-mutant) and NCH89 (human short-term glioblastoma culture) cell lines (Table 1). Various subtoxic daidzein concentrations (50–200 μM), when treated with those cells, caused a significant reduction of cell viability, with promoted apoptotic cell death. Key findings included downregulation of B-cell lymphoma 2 (Bcl-2), the anti-apoptotic protein, along with activation of caspase-9, which stimulated cell death rate. The expression of various death receptors, such as cellular Fas-associated death domain protein (FADD)-like IL-1β-converting enzyme (FLICE)-inhibitory protein (c-FLIP), X-linked inhibitor of apoptosis protein (XIAP), and survivin, remained unchanged, hinting toward the intrinsic apoptotic pathway. Another study by Praisthy et al. [62] demonstrated the importance of daidzein in modulating phosphatidylinositol 3-kinase (PI3K)-protein kinase B/Ak strain transforming (Akt) pathway, crucial for the pathogenesis of glioblastoma. At various concentrations (100–1000 μg/mL), daidzein was incorporated against the SH-SY5Y human neuroblastoma cell line, and a significant reduction of cell viability was inferred. Moreover, marked antioxidant potency of daidzein was concluded, as significant elevation of superoxide dismutase (SOD) and reduced glutathione (GSH) with aberration of thiobarbituric acid-reactive substances (TBARS) levels were observed post-treatment.

A promising recent work by Li et al. [63] also established the anticancer effect of daidzein in glioma cells. A marked aberration of cellular viability, coupled with increased apoptotic rate, was the main inference drawn when daidzein at two different concentrations (50 and 100 μM) was applied against U251 glioma cells. Major morphological changes, such as pyknosis, were observed in the daidzein-treated cells. Expression of various mRNA was altered, specifically, upregulation of Bcl-2-associated X protein (Bax) and caspase-3, followed by downregulation of phosphorylated Akt (p-Akt) and phosphorylated mammalian target of rapamycin (p-mTOR).

### 3.2. Breast Cancer

Breast cancer is the second most common cancer in women after skin cancer [64]. It occurs as a neoplastic growth developing from mammary gland tissue and can metastasize locally in the breast and outside it. Even though breast cancer affects mostly women, it can also affect men. Clinical manifestations frequently include the presence of a palpable breast lump, modification in breast texture or shape, and occasionally, bleeding nipple discharge [65].

In one study [66], daidzein was found to be oncopreventive in nature for both estrogen receptor (ER)-positive MCF-7 and epidermal growth factor receptor 2 (ErbB2)-overexpressing MDA-MB-453 human breast cancer cell lines. Daidzein (1–100 μM, 24–72 h) profoundly inhibited proliferation in a time- and concentration-dependent manner, with increased potency in the MDA-MB-453 cell line. Flow cytometric profiling showed G_1_ and G_2_/M phase cell cycle arrest at ≥5 μM in MCF-7 and ≥10 μM in MDA-MB-453 cell lines after 72 h. In addition, daidzein enhanced sub-G_0_ apoptotic fractions accumulation in MDA-MB-453 cell lines, but not in MCF-7 cell lines. At the molecular level, daidzein suppressed cyclin D, cyclin-dependent kinase (CDK) 2, and CDK4, with significantly low CDK1 expression. Parallel to this, it upregulated CDK inhibitors p21Cip1 and p57Kip2, indicating dual regulation of G_1_ and G_2_/M checkpoints. Caspase-9 activity was significantly enhanced as well, incriminating intrinsic apoptotic signaling. In another study [67], daidzein was examined for growth inhibitory and pro-apoptotic activity in MCF-7 breast cancer cells. Exposure for 24–72 h to daidzein (25–100 μM) inhibited cell viability in a concentration- and time-dependent fashion. Flow cytometry and Hoechst–propidium iodide (PI) staining showed intense induction of apoptosis, with apoptotic levels reaching 29.8% at 100 μM following 24 h. Mechanistic investigations proved daidzein-induced ROS production, disrupted mitochondrial membrane potential, downregulated anti-apoptotic Bcl-2, and upregulated Bax. The cascade proceeded through cytochrome c (cyt. c) release, caspase-7 and caspase-9 activation, and consequent apoptosis. Treatment with the antioxidant NAC and the pan-caspase inhibitor z-VAD-fluoromethylketone (FMK) reduced apoptosis, verifying participation of the mitochondrial caspase-dependent pathway.

Another study compared daidzein’s antiproliferative activity in three different breast cancer cell lines with varying receptor profiles: ERα-positive MCF-7, ErbB2-positive SKBR-3, and ERα/ErbB2-positive ZR-75-1. Following 72 h treatment (1–200 μM), daidzein displayed biphasic effects in the MCF-7 cell line, promoting proliferation at low concentrations (1 μM) and suppressing proliferation at higher doses, with the 24.64% inhibition at 200 μM. Conversely, SKBR-3 and ZR-75-1 cell lines showed concentration-dependent inhibition, where the half maximal inhibitory concentration (IC_50_) values were 211.7 μM and >200 μM, respectively. Daidzein repressed ERα and ErbB2 expression in SKBR-3 and ZR-75-1 cell lines and caused apoptosis in the ZR-75-1 cell line, as established by 4′,6-diamidino-2-phenylindole (DAPI) staining [68]. The invasive capacity of triple-negative breast cancer cells has also been found to be suppressed by daidzein. The suppression of cell invasion through Matrigel by 50–60% was achieved in the MDA-MB-231 cell line by treatment with daidzein at concentrations of 2.5, 10, and 50 μM for 48 h. The suppression was correlated with the reduction in matrix metalloproteinase (MMP)-2 expression, but no changes were seen in MMP-9, tissue inhibitors of metalloprotease (TIMP)-1, or TIMP-2 expression. Comparable anti-invasive activities were seen with R- and S-equol, suggesting equipotent activity of daidzein and its metabolites to inhibit the invasion of invasive breast cancer cells [69].

The function of daidzein in regulating cytokine-induced invasion was investigated in the ER-negative basal-like MCF10DCIS.com cell line. Treatment with tumor necrosis factor-α (TNF-α) at a concentration of 5 ng/mL provoked extensive migration and invasion, which were remarkably suppressed by daidzein (30 μM, 24 h). Mechanistically, TNF-α stimulated the Hedgehog (Hh) pathway through increased nuclear translocation of glioma-associated oncogene homolog 1 (Gli1) and elevated transcriptional activity, and daidzein treatment inhibited Gli1 activation. Concurrently, daidzein inhibited TNF-α–induced MMP-9 expression and activity, involved in invasion suppression. These results identify daidzein as a potential inhibitor of inflammatory cytokine-mediated invasion through the Hh/Gli1 axis targeting [70]. A research work by Koo et al. [39] assessed the anticancer activity of daidzein against breast cancer cells. In vitro, incubation of the ER-positive MCF-7 cell line with daidzein (3 and 10 μM, 48 h) caused remarkable inhibition of cell growth, with greater inhibition at the higher concentration. The study also inferred decreased viability of another cell line, i.e., ER-negative MDA-MB-231, upon treatment with 10 μM (48 h) daidzein. These antiproliferative actions persisted despite the simultaneous induction of butyrate response factor (BRF)1, BRF2, and ribonucleic acid (RNA) polymerase III transcripts, which are typically associated with driving oncogenic signaling. Mechanistic examination showed that daidzein worked by stabilizing BRF1 and BRF2 messenger RNAs (mRNAs) and selective demethylation of the BRF2 promoter, but had a net biological effect of growth inhibition, validating daidzein’s anticancer activity in ER-positive and ER-negative breast cancer cell lines.

Daidzein has also been studied for its modulatory action on estrogen-mediated survival signaling pathways in ER-positive breast cancer cells. Exposure of MCF-7 and T47D cell lines to daidzein (1–10 μM) for 24 h dramatically decreased the expression level of neuroglobin (NGB), a protein that is inducible by ERα and is anti-apoptotic. This action was replicated by its sulfate metabolite daidzein-4′-sulfate (D4S), whereas equol and other metabolites enhanced NGB expression in a pattern comparable to estradiol. Daidzein and D4S downregulated NGB, and this was linked to ERα activation since pre-treatment with ERα inhibitor endoxifen abolished these actions. In addition, daidzein and D4S sensitized MCF-7 and T47D cell lines to paclitaxel-induced apoptosis, indicating that these compounds disrupt the estrogen/ERα-dependent anti-apoptotic pathway and augment chemotherapeutic potency [71]. In another in vitro study, it has been shown that daidzein has concentration-dependent activity against breast cancer cells. Daidzein treatment of ER-positive MCF-7 cell line at concentrations of 25–100 μM for 24–72 h suppressed proliferation time- and concentration-dependently, with an IC_50_ value of 50 μM. Apoptosis was verified by Annexin V staining and an increase in caspase-3/7 activity, which was 1.4-fold higher than untreated controls after treatment for 48 h with 50 μM. Mechanistically, daidzein induced the mitochondrial apoptotic pathway, which was evidenced by Bax upregulation, Bcl-2 downregulation, and enhanced ROS production. Notably, daidzein treatment changed the ratio of ERs such that there was decreased ERα and enhanced ERβ, thus reducing the ERα/β ratio, which is a positive apoptotic signaling indicator [72]. An analysis by Zubair et al. [73] assessed the anticancer activity of daidzein against breast cancer cells. Daidzein showed strong cytotoxicity with an IC_50_ of 0.04 µg/mL in ER-positive T47D cells after 48 h of incubation, showing higher activity than that of quercetin. Significantly, the compound was highly selective by showing little cytotoxicity in non-tumorigenic Vero cells. In the same study, molecular docking and dynamics simulations predicted that daidzein will bind to epidermal growth factor receptor tyrosine kinase (EGFR-TK) with a docking score of −9.6 kcal/mol. However, these in silico analyses are hypothesis-generating only and do not indicate whether daidzein actually binds to the target receptor or if it has any binding affinity to that target in biological cells. Experimental validation of these predictions would be required to determine if EGFR signaling plays any role in the effects of daidzein that are observed in those cells.

Newly devised therapy has targeted daidzein as an enzyme-directed prodrug therapy targeting moiety. A daidzein-conjugated methionine γ-lyase (C115H-Dz) was designed to selectively target ER-positive MCF-7, SKBR-3, MDA-MB-231, and T-47D breast cancer cells. This enzyme depletes extracellular methionine and catalyzes the conversion of S-(allyl/alkyl)-L-cysteine sulfoxides to the cytotoxic dipropyl thiosulfinates. Potent cytotoxicity was observed with an IC_50_ of less than 0.53 mM after 72 h of treatment [74]. A recent study proved that daidzein causes selective ferroptosis in triple-negative breast cancer cells. Exposure of MDA-MB-231 and MCF-7 cell lines to daidzein for 48 h led to concentration-dependent toxicity with IC_50_ values of 25.36 µM and 33.23 µM, respectively, whereas significantly less toxicity was noted in non-tumorigenic MCF-10A cells (IC_50_ = 154.7 µM). Treatment with ferrostatin-1 (1 µM) strongly reversed daidzein’s cytotoxicity in the MDA-MB-231 cell line but not in the MCF-7 cell line, affirming that ferroptosis was the prevalent form of cell death in triple-negative cells while apoptosis was more applicable in the ER-positive MCF-7 cell line, evidenced by partial rescue with Z-VAD-FMK (20 µM). Mechanistically, daidzein promoted lipid peroxidation and intracellular Fe^2+^ content and decreased the GSH/glutathione disulfide (GSSG) ratio in MDA-MB-231 cell line, with a concomitant large downregulation of anti-ferroptotic regulators GPX4 (−48.1%) and ferroptosis suppressor protein (FSP)-1 (−24.7%) at the mRNA level. In contrast, GPX4 and FSP-1 expression did not change in the MCF-7 cell line, where ferroptosis did not play a role. The above findings indicate that daidzein has the potential to influence ferroptosis-related processes in TNBC cells. However, these conclusions are based only on in vitro studies. Further studies are therefore required to investigate whether ferroptosis is the main mechanism of action of daidzein’s anticancer effects [75].

In vivo chemopreventive activity of daidzein was assessed in a rat model of chemically induced mammary carcinogenesis. The study involved oral treatment with daidzein (0.69–6.9 mg/kg/day, equivalent to 2.7–27 μmol/kg/day) for 3 weeks, which inhibited the development of 7,12-dimethylbenz[a]anthracene (DMBA)-induced mammary tumors in Sprague Dawley rats (Table 2). Tumor development was decreased to 2.9-fold relative to an 8.1-fold increase in controls, and apoptosis was increased to 25% by flow cytometry. Equol, the predominant metabolite yielded from daidzein, had even greater tumor suppression, but tamoxifen at the same molar dose did not have a significant inhibitory effect. Simultaneously, in ovariectomized athymic nude mice bearing MCF-7 xenografts and estrogen pellets, tumor growth was more potently inhibited by oral daidzein (6.9 mg/kg/day for 4 weeks) than by tamoxifen. Immunohistochemistry validated augmented caspase-3 expression within tumor tissue, lending credence to the induction of apoptosis as a primary mechanism [40]. In a separate study, Koo et al. [39] showed that daidzein has sex-dependent action on BRF expression in mice. High-isoflavone-fed female C57BL/6J mice, for 3 weeks, showed increased mRNA levels of BRF2, indicating a possible oncogenic response. Importantly, the finding of upregulation of BRF2 is also associated with tumor-promoting activity, which could negate the anticancer effects that daidzein is otherwise known to exhibit in certain contexts. Male mice fed with the same diet presented marked suppression of both BRF1 and BRF2 expression, suggesting a protective anticancer effect. These findings are critical to understanding daidzein as they suggest that the effects of daidzein may differ between sexes. Thus, the potential for daidzein to exhibit tumor-promoting effects in the female sex and populations with high estrogen levels should be considered in the interpretation of these studies and any future studies of daidzein.

### 3.3. Cervical Cancer

Cervical cancer is a type of malignancy that arises in the cells of the cervix, which constitutes the lower part of the uterus extending to the vaginal canal. Its development results from genetic changes or mutations within the deoxyribonucleic acid (DNA) of otherwise normal cervical cells and results in unlimited growth and cancerous transformation. Cervical cancer histologically falls into two primary categories: squamous cell carcinoma and adenocarcinoma [76].

Daidzein has been shown to exhibit cytotoxic activity against different cervical cancer cell lines. One of the first studies investigated the impact of daidzein on the human cervical cancer HeLa cell line. Daidzein treatment (6.25–100 µM, 24–96 h) markedly suppressed cell growth in a time- and concentration-dependent manner. Flow cytometry analysis indicated that exposure to daidzein resulted in cell cycle arrest in both G_0_/G_1_ and G_2_/M phases based on concentration and duration, resulting in a decrease in the S phase population. In the lower concentrations (6.25–12.5 µM), daidzein triggered apoptosis as evidenced by the rise in sub-G_0_/G_1_ peak. Mechanistic analysis proved that treatment with daidzein inhibited the expression of human telomerase reverse transcriptase (hTERT) mRNA in the HeLa cell line, which was an indicator of inhibition of telomerase activity. These results established that daidzein growth inhibitory activity against cervical cancer cells involves the regulation of cell cycle progression, induction of apoptosis, and inhibition of telomerase activity [77]. Daidzein suppressed cell growth in the HeLa cell line with an IC_50_ of 0.54 µg/mL after 48 h of incubation, demonstrating greater cytotoxicity than quercetin. As in the case of breast cancer, daidzein was highly selective and caused minimal or no toxicity in normal Vero cells. In silico studies helped to validate the stable association of daidzein with EGFR-TK. Similar to the results for breast cancer, these studies did not provide evidence of a target for daidzein without further studies to confirm such a finding [73].

In another study, Yao et al. [78] explored the anticancer potency of the isoflavone phytoestrogen daidzein against the human cervical cancer HeLa cell line. Through the 3-[4,5-dimethylthiazol-2-yl]-2,5-diphenyl tetrazolium bromide (MTT) assay, daidzein was found to possess substantial cytotoxicity with an IC_50_ of 20 μM (24 h), which was then used for further mechanistic studies. Daidzein treatment enhanced intracellular ROS generation, damaged mitochondrial membrane potential (seen through rhodamine-123 staining), and triggered apoptosis as established by acridine orange/ethidium bromide (AO/EtBr) staining. Additionally, cell adhesion was inhibited through Matrigel assays, implying its potential to curtail metastatic capability. Induction of apoptosis was also established by the enhancement of caspase-8 and caspase-9 activity, indicating involvement of both extrinsic and intrinsic pathways of apoptosis. Furthermore, quantitative polymerase chain reaction (qPCR) analysis showed a significant repression of proliferative and inflammatory signaling molecules, emphasizing the modulation of oncogenic signaling by daidzein. Overall, these results indicate that daidzein possesses strong anticancer activities in the HeLa cell line through the induction of oxidative stress, activation of caspase-mediated apoptosis, and suppression of pro-inflammatory and proliferative pathways, validating its potential as a natural therapeutic compound for cervical cancer.

A recent report assessed the anticancer activity of daidzein in the HeLa cervical cancer cell line. Daidzein treatment (20–40 µM, 48 h) inhibited colony formation and downregulated proliferation markers minichromosome maintenance protein 2 (MCM2) and proliferating cell nuclear antigen (PCNA). Flow cytometric analysis confirmed that daidzein caused cell cycle arrest at the G_1_ phase, with a corresponding upregulation of p21 and downregulation of cell cycle regulators such as cell division cycle 25C (Cdc25C), CDK1, and cyclin B1. In addition, daidzein-induced apoptosis through the intrinsic mitochondrial pathway, as illustrated by disruption of mitochondrial membrane potential, enhanced cleavage of caspase-3 and poly (ADP-ribose) polymerase (PARP), and inhibition of anti-apoptotic proteins B-cell lymphoma-extra-large (Bcl-xL) and survivin. These alterations are commonly seen in cells under stress or undergoing apoptosis and are not necessarily indicative of a daidzein-specific mechanism. In addition, daidzein suppressed the migration and invasion of the HeLa cell line by downregulating urokinase plasminogen activator receptor (uPAR) and N-cadherin expression, while simultaneously inhibiting STAT3 phosphorylation and c-Jun N-terminal kinase (JNK) signaling activation. These observations highlight the potential of daidzein to suppress proliferation, induce apoptosis, and disrupt metastatic behavior in cervical carcinoma cells [79].

### 3.4. Choriocarcinoma

Choriocarcinoma is best defined as the most common form of gestational trophoblastic neoplasia, which occurs from the malignant transformation of stem cells present within the trophoblast [80]. The major cells formed are known as cytotrophoblast, which are later on dedifferentiated into syncytiotrophoblasts or intermediate trophoblasts. This form of carcinoma commonly affects women during gestation, though it can also be observed in postmenopausal women [81,82,83].

In a study by Zheng et al. [41], the antineoplastic activity of daidzein against JAR and JEG-3 choriocarcinoma cell lines was evaluated in vitro. Reduced cell proliferation and cell cycle arrest at the G_1_ phase were among the primary observations from the study. The anticancer effects of daidzein were attributed to the upregulation of p21 genes and attenuated expression of myelocytomatosis oncogene (c-Myc), cyclin D1, PCNA, and phosphorylated extracellular signal-regulated kinase (p-ERK), as confirmed from the Western blot analysis. Zheng et al. [84] further discovered daidzein’s anticarcinogenic activity against JAR and JEG 3 human gestational choriocarcinoma cells, following the mitochondrial damage-mediated apoptotic pathway. Daidzein at various concentrations (12.5–400 µM) was able to inflict mitochondrial damage, as activation of caspase-3, caspase-9, and PARP, with reducing Bcl-2/Bax ratio, was primarily inferred from the Western blot test results. Furthermore, from the MTT assay, it was observed that cellular viability was diminished as the cellular death rate was increased exponentially.

In vivo study of xenografting JEG-3 cells subcutaneously (*s.c.*) in male BALB/c nude mice was performed by Zheng and colleagues [41]. Tumorigenesis was studied pre- and post-treatment of daidzein, and from the studies, it was concluded that the respective soy isoflavone has the potential to reduce tumor growth and volume as well as reduce the incidence of tumor occurrence. The signaling pathway involved in such observations was considered to be downregulated PCNA, p-ERK, and c-Myc levels, which were confirmed from various analytical procedures.

### 3.5. Colon Cancer

Colon cancer or colorectal cancer is a term applied to malignancies in the colon and the rectum, which is the terminal part of the large intestine. The disease usually starts with the occurrence of small, harmless cell clusters termed as polyps on the inner wall of the colon. Some of these polyps, over the years, could undergo malignant changes and cause colon cancer [85,86].

Guo et al. [87] studied daidzein’s impact on the human colorectal carcinoma LoVo cell line. The cell line was exposed to daidzein between 0.1 and 100 μM for 48–120 h. The effect was biphasic in nature, with low concentrations (0.1–1 μM) inducing cell proliferation and high concentrations (10–100 μM) inhibiting it concentration-dependently. Flow cytometric analysis demonstrated G_0_/G_1_ phase arrest at 10–100 μM, while the sub-G_1_ apoptotic peak appeared. DNA fragmentation assays verified apoptosis, while caspase-3 activity was drastically increased after exposure to 10–100 μM daidzein. Conversely, alkaline phosphatase activity did not change, suggesting that differentiation was not implicated in the growth inhibition observed. These findings demonstrate that daidzein inhibits LoVo cell growth mainly by cell cycle arrest and apoptosis induced by caspase-3 activation, but not by inducing differentiation. Lepri et al. [88] assessed the antiproliferative activity of daidzein on the HT-29 human colon adenocarcinoma cell line. Daidzein treatment (10–100 µM, 24–96 h) suppressed proliferation in a time- and concentration-dependent fashion, with viable cell reduction by 48% at 100 µM after 96 h. Reverse transcription-PCR analysis demonstrated that daidzein (10 or 50 µM, 12 h) failed to cause a significant change in the transcript levels of β-catenin, adenomatous polyposis coli (APC), or survivin, as compared to genistein, which inhibited β-catenin expression. It indicates that daidzein produces weak growth inhibitory activity on colon cancer cells, though its action on wingless-related integration site (Wnt)/β-catenin signaling might be restricted when compared to other isoflavones.

Another study using HT-29 human colorectal adenocarcinoma cells, Liang et al. [89] showed that daidzein (25–400 µM, 48 h) suppressed proliferation and caused apoptosis in a concentration-dependent manner. The process entailed inhibition of lipid droplet accumulation and downregulation of perilipin-1, adipophilin/adipose differentiation-related protein, tail-interacting protein 47, and vimentin proteins. Daidzein activated Forkhead box O (FOXO)3a and caspase-8 and decreased PI3K expression, indicating that apoptosis was being mediated through PI3K/FOXO3a/caspase-8 signaling and modulation of the peroxisome proliferator-activated receptor (PPAR)-γ pathway, hence linking lipid droplet metabolism with apoptotic regulation in colon cancer cells. Gundogdu et al. [90] studied the cytotoxicity and genotoxicity of daidzein in the HT-29 colorectal adenocarcinoma cell line. Exposure to 200 µM daidzein for 48 h produced significant inhibition of cell viability by the 2,3-bis-(2-methoxy-4-nitro-5-sulfophenyl)-2H-tetrazolium-5-carboxanilide (XTT) assay. Genotoxicity was established by the comet assay, which showed extensive DNA strand damage in the daidzein-treated cell line. Particularly, DNA tail moment and DNA tail intensity were markedly higher than in controls, which reflects enhanced DNA fragmentation. These findings confirm that daidzein has both cytotoxic and genotoxic effects in the tested colorectal cancer cell line in a time- and concentration-dependent fashion, mainly through the induction of DNA damage.

In another experiment, Salama and Allam [42] analyzed the anticancer activity of daidzein on the SW620 human colon cancer cell line. The cell line was exposed to daidzein at concentrations of 10–50 µM for 48 h. Treatment resulted in the inhibition of phosphorylation of ERK and Akt, two key kinases of the EGFR-mediated signaling pathway that control growth, survival, and invasion. By blocking such oncogenic signaling pathways, daidzein decreased molecular drivers implicated in aggressive tumor behavior in colorectal cancer cells. These results indicated that daidzein is able to directly target intracellular signaling to inhibit malignant potential in vitro.

Salama and Allam [42] examined the in vivo chemopreventive activity of daidzein against colorectal cancer through a combined 1,2-dimethylhydrazine (DMH)/dextran sulfate sodium (DSS) rat model. Rats received 1,2-dimethylhydrazine (DMH, 40 mg/kg/week, *s.c.* for 16 weeks) along with DSS for initiating colorectal carcinogenesis. Oral administration of daidzein at 5 and 10 mg/kg doses, three times a week for 8 weeks, significantly suppressed tumor development. Biochemical profiling showed that daidzein decreased systemic levels of circulating amphiregulin (AREG), chemokine ligand (CXCL1), and MMP-9, the central mediators of tumor growth, inflammation, and invasion. Concurrent with this, daidzein increased antioxidant defense by inhibiting cytochrome P-450 (CYP) 2E1 activity, indicating preservation against oxidative stress-induced DNA damage. Histopathological analysis validated these biochemical results and demonstrated reversal of mucosal hyperplasia, dysplasia, and inflammatory infiltration with restoration to normal colonic tissue architecture. The outcomes proved that daidzein has strong chemopreventive action in vivo by inhibiting inflammatory signaling, downregulating tumor-promoting mediators, and safeguarding the integrity of the colon tissue.

### 3.6. Gastric Cancer

Among various gastrointestinal malignancies, gastric cancer is considered the fifth most common form of cancer globally [91]. This carcinoma arises from neoplastic growth within the gastric mucosa, subsequently invading various layers of the stomach. Dietary lifestyle, family history, epigenetic alterations, and different microorganism infections, such as *Helicobacter pylori* and Epstein–Barr virus, are considered to be the major epidemiological factors responsible for the prognosis of this disease [92,93].

Several studies have been conducted to comprehend the anticarcinogenic activity of daidzein against gastric cancer. Tang and his colleagues in 2013 performed an in vitro study to comprehend the anti-gastric cancer efficacy and to ameliorate the mechanistic pathway involved. Daidzein at various concentrations (20–80 μM) inhibited BGC-823 gastric cancer cellular proliferation and viability. The cytotoxic potential of daidzein against the cell line was further supported with an apoptosis assay and flow cytometry, respectively. In the Western blot analysis, upregulation of cleaved caspase-3, caspase-9, and PARP, with significant downregulation of Bcl-2/Bax suggested the mechanistic pathway involved for the respective therapeutic effects [94]. A more recent study by Ge et al. [95] also concluded similar types of effects of daidzein against BGC-823 gastric cancer cell lines. Cellular proliferation was minimized by daidzein at a concentration of 29.90 μM (IC_50_), and enhanced ROS generation influenced the cell death process. Cell cycle arrest at the G_0_/G_1_ phase with increased apoptotic death was also a major inference drawn from the study. Suppression of signal transducer and activator of transcription 3 (STAT3) and focal adhesion kinase (FAK) phosphorylation directly aberrated the expression of cyclin-D1, Bcl-2, and MMP-2, which are crucial survival markers of gastric carcinoma.

### 3.7. Head and Neck Cancers

Head and neck squamous cell carcinomas (HNSCC) are a group of malignancies affecting the oral cavity, pharynx, and larynx, with high morbidity and mortality rates [43].

Recent research has begun to uncover the therapeutic potential of daidzein in this challenging cancer type. Mao et al. [43] investigated the role of daidzein in hypopharyngeal squamous cell carcinoma (HPSCC), a lethal malignancy with limited treatment options. Using a multi-omics approach, the researchers identified that loss-of-function mutations in the histone acetyltransferases E1A-binding protein p300 (EP300) and CREB-binding protein (CREBBP) are key drivers of HPSCC progression. These mutations lead to impaired histone acetylation and suppression of PPARγ, subsequently activating the oncogenic angiopoietin 4 (ANGPT4)/tyrosine kinase with immunoglobulin-like and EGF-like domains 2 (Tie2) signaling axis and reshaping the immune microenvironment toward an immunosuppressive, regulatory T cell (Treg)-dominant profile. Strikingly, treatment with daidzein (20–40 mg/kg intraperitoneally in a murine xenograft model) effectively restored histone acetylation and PPARγ expression. This led to the inhibition of ANGPT4/Tie2 signaling, reduced tumor growth and Ki-67 expression, and reversed immune evasion by lowering immunosuppressive cytokines, such as transforming growth factor-β (TGF-β) and IL-35. The study positions daidzein as a dual agonist of EP300 and PPARγ, offering a novel therapeutic strategy for epigenetic defects in HPSCC.

### 3.8. Liver Cancer

Hepatocellular carcinoma is considered the sixth most common and deadliest variant of cancer, occurring globally, with a less than 20% survival rate [91]. Hepatocellular cancer is principally associated with non-communicable risk factors, including chronic liver disease, non-alcoholic fatty liver disease, and alcohol induced liver cirrhosis, followed by hepatitis B and hepatitis C as communicable risk factors. Immunomodulatory modifications and epigenetic alterations also play a crucial role in the pathogenesis of this disease [96,97,98].

In an in vitro study by Park et al. [99], daidzein was found to exhibit a potent antineoplastic effect against the SK-HEP-1 hepatocellular carcinoma cell line. The preliminary findings suggested that daidzein induces apoptotic cell death, leading to reduced viability of the carcinoma cells. The mechanisms involved in the death of the carcinoma cells include DNA damage and ROS generation. Western blot analysis, highlighting the balance of markers required for viability of cancer cells, depicted downregulation of Bcl-2, Bcl-xL, and Puma with significant activation of caspase-3, caspase-7, and caspase-9, causing cyt. c-mediated apoptotic changes in the cell. Han et al. [100] used various cell lines of different sorts of carcinomas to evaluate the cytotoxic potency of daidzein in vitro. Significant cytotoxicity against BEL-7402 cells was inferred along with cell cycle arrest at G_2_/M phase. Additionally, ROS-mediated apoptosis was also observed in daidzein-treated cells, supported by downregulated Bcl-2, Bcl-xL, and BH3-interacting death domain agonist (Bid) proteins, with marked upregulation of Bcl-2 interacting mediator of cell death protein. Li et al. [44] investigated the effect of daidzein against the migration and viability of HCCLM3 and Hep3B human hepatoma cell lines. Marked reduction in cell survivability and migration was inferred from the study, due to stimulation of Bax and PARP, followed by consequent inhibition of CDK1, c-Myc, Bcl-2, and survivin. Moreover, alteration in the glycolysis/gluconeogenesis pathways as an effect after daidzein treatment was also observed. The key mechanistic pathway involved was downregulation of TPI1, a gene in the glycolysis pathway, which is majorly required for the hepatocellular carcinoma cells for maintaining viability.

Similar to the in vitro studies, many in vivo experiments demonstrating the anti-hepatocellular carcinoma effect of daidzein have also been performed. Li and his colleagues [44] first investigated the anticancer activity of daidzein against liver cancer by *s.c.* administering Hep3B cells in female nude mice. Application of daidzein in treatment groups caused a marked reduction in tumor weight and volume, followed by successful aberration of Ki-67 expression in the formed tumors. Bashandy et al. [45] investigated the anticancer activity of daidzein against diethylnitrosamine (DENA)/carbon tetrachloride (CCl4) induced hepatocellular carcinoma in male adult Wistar rats. Different doses of daidzein were administered to evaluate the anticancer effect, among which 40 mg/kg was able to normalize elevated hepatic biomarkers, such as alkaline phosphatase, alanine aminotransferase, and aspartate aminotransferase. Moreover, carcinogenic biomarkers such as alpha-fetoprotein, glypican-3, and vascular endothelial growth factor (VEGF), along with some inflammatory markers, such as IL-6, TNF-α, and C-reactive protein (CRP), which were elevated post-administration of DENA/CCl4, were significantly lowered after treatment. Moreover, histological studies have reported improvement in morphological characteristics, thereby proving daidzein’s anti-hepatocellular carcinoma potential.

### 3.9. Lung Cancer

Lung cancer is considered one of the deadliest forms of cancer, ranked as the seventh deadliest type of carcinoma, affecting patients from both the smoking and non-smoking communities [101]. It is characterized by neoplasm growth in the lungs and the respiratory tract. Several factors, such as lifestyle, environmental elements, and harmful chemical exposure, drive the prognosis of this form of carcinoma. The main mechanisms involved in the pathogenesis of lung cancer are either overexpression of inflammatory biomarkers or epigenetic modifications, after exposure to a harmful environment [102,103].

Among the various in vitro investigations, Chen et al. [104] conducted the first research to evaluate the anti-lung cancer potential of daidzein. Two lung carcinoma cell lines, namely A549 and H1299, were exposed to various concentrations of daidzein to observe the antineoplastic effect. Daidzein was able to halt cellular proliferation, as a greater proportion of cell death was observed as a result of apoptosis. Additionally, daidzein restored serine/threonine-protein kinase 4 (STK4) levels and increased the phosphorylation of yes-associated protein 1 (YAP1) and overexpression of cleaved caspase-3, as assessed by RNA interference technique, contributing to the mechanistic pathway involved in apoptotic cell death. Guo et al. [46] also contributed to establishing the anti-lung carcinoma effect of daidzein, as evaluated against A594 and 95D cells. Marked inhibition of the cellular proliferation was observed, due to active reduction of IL-6 and IL-8 formation in the treated cells. Daidzein also attenuated the expression and activation of NF-κB, which contributed to aberrant cellular proliferation.

Another study by Liu et al. [105] concluded a similar type of antineoplastic effect against lung carcinoma when evaluated against H1299 lung adenocarcinoma cells. Treatment of those cells with daidzein at a concentration of 10 µM was able to induce apoptotic cell death by stimulating the proapoptotic genes such as tumor protein 53 (TP53) and caspase-9. Moreover, the addition of pifithrin-α (PFTα) to H1299 cells caused downregulation of TP53 genes, but they were increased exponentially post-treatment of daidzein, proving the apoptotic action of this isoflavone. Li et al. [106] utilized an innovative approach in comprehending the anti-lung cancer effect of daidzein, when expression of various long non-coding RNAs (lncRNAs) in H1299 cells was assessed using microarray analysis post-treatment with the respective isoflavone. The inferences were upregulation of eight and downregulation of 111 lncRNAs, accompanied by overexpression of five mRNAs and aberrated expression of 35 mRNAs, that halted the proliferation of the lung adenocarcinoma cell line. Expression of several lncRNAs, such as ENST00000608897.1 and ENST00000444196.1, was promoted, followed by downregulation of XR_242163.1. Additionally, mRNAs such as rhophilin-associated tail protein 1-like (ROPN1L) and Fanconi Anemia complementation group C (FANCC) were overexpressed in treated H1299 cells, with marked aberration of thyrotropin-releasing hormone receptor (TRHR) and insulin-like growth factor 1 (IGF1) levels.

Alterations in various signaling pathways have also led to decreased cancer cell growth and progression. In a study by Mhone et al. [47], it was found that daidzein at different concentrations actively inhibited A549 and H1975 cell proliferation and induced apoptosis by directly inducing c-Jun nuclear translocation through ROS/apoptosis signal-regulating kinase 1 (ASK1)/JNK and dampening epidermal growth factor receptor (EGFR)—signal transducer and activator of transcription (STAT)/Akt/ERK pathways. Modifications in cell cycle regulators, such as upregulation of p53, followed by marked downregulation of p27, p21, and p16, caused abrupt cell cycle arrest at the G_0_/G_1_ phase. Among the various members of the transmembrane protein family, a particular protein, i.e., transmembrane protein 16A (TMEM16A)/calcium-activated chloride channels (CaCCs), can be considered the most dominant one, abundantly expressed on lung cancer cells, participating in cell viability and proliferation. Wang et al. [48] inferred from their study that daidzein (IC_50_ of 1.39 ± 0.59 μM) concentration-dependently inhibited both expression of TMEM16A protein and current, as evaluated in LA795 cells. Further molecular docking studies have identified the binding sites of daidzein in the specific protein, especially at G608, G628, and K839. Other important findings were aberrant cell viability and migration, with cell cycle arrest at G_1_/S phase.

Menon et al. [49] conducted the first research in vivo, aiming to justify the anti-lung cancer effect of daidzein. The tumorigenic model was produced by xenografting B16F-10 melanoma cells in C57BL/6 male mice. Daidzein, with a dose of 200 μmol/kg body weight, was meticulously able to reduce lung tumor volume as well as aberrant serum sialic acid and lung hydroxyproline content, as compared to the diseased group. Histopathological investigations have also signified improved morphological characteristics of lungs and alveolar tissues. Guo and his colleagues [46] also performed an in vivo study to validate the antineoplastic effect by applying daidzein in A594 xenografted tumors in BALB/c nude mice. Daidzein was able to reduce the tumor growth by directly attenuating the expression of p65-NFκB and Ki-67 in the tumor xenografts. Apoptotic cell death in A549 xenografted tumor in male nu/nu nude mice post-treatment of daidzein in vivo was also reported by Mhone et al. [47], as a direct effect of ASK-1/JNK pathway induction and cleaving PARP-1 and caspase-3, confirmed by Western blot analysis.

A novel lung carcinoma model, as established by Feng et al. [50], comprised male Swiss albino mice treated with benzo(a)pyrene orally, which were treated with daidzein (20 mg/kg) to validate the anti-lung carcinoma effect. Daidzein was able to reduce tumor growth and incidence, along with reducing immunoglobulin-A (IgA), followed by overexpressing IgG and IgM serum levels. Moreover, several tissue markers, such as aryl hydrocarbon hydroxylase (AHH), adenosine deaminase (ADA), lactate dehydrogenase (LDH), and γ-glutamyl transferase (GGT), along with pro-inflammatory cytokines, including IL-1β, IL-6, and TNF-α, which were elevated after inducing the carcinogenic chemical, were significantly down-streamed. Lastly, various factors that participate in tumor cell proliferation, namely PCNA, CYP1A1, NF-κB, and Nrf2, whose levels were markedly elevated during lung cancer progression, were reduced to a massive extent, proving the antineoplastic effect of this isoflavone. Wang et al. [48] also investigated the anti-lung cancer effect of daidzein by establishing a novel model through *s.c.* injection of LA795 cells in BALB/c mice. After application of daidzein to the experimental model, marked abolition of tumor growth and volume was observed, without causing any significant toxic change to major organs.

### 3.10. Osteosarcoma

Osteosarcoma is best defined as a heterogeneous malignancy in the bones and other associated soft tissues, primarily affecting children and adolescents [107]. The crucial characterization of this carcinoma is the presence of several immature cells, having a potent, aggressive phenotype and uncontrolled cellular proliferation [108,109]. An abnormal tumor microenvironment is formed, which facilitates tumor growth and progression, ultimately demineralizing the bones and affecting the structural integrity [110,111].

Zhu et al. [51] first investigated the in vitro antineoplastic effect of daidzein against osteosarcoma by evaluating its potency against 143B and U2OS cell lines. Daidzein was able to inhibit the cellular proliferation of 143B (IC_50_: 63.59 µmol/L) and U2OS (IC_50_: 125 µmol/L) osteosarcoma cells. Additionally, cellular death by apoptosis and aberration of the cell cycle at the G_0_/G_1_ phase also proved the anticancer activity of the respective isoflavone, which was triggered by downregulating phosphorylation of Src, which is a proto-oncogene, and ERK, which ultimately aberrated the MAPK pathway. Another study by Wang et al. [52] demonstrated the application of daidzein against a variety of osteosarcoma cell lines, mainly comprising U2OS, HOS, MNNG/HOS, SJSA-1, 143B, and MG63 cells. Marked reduction in cellular viability and stimulated apoptosis rate were the prime mechanistic pathway modulations responsible for the observed antineoplastic effects. Additionally, downregulation of β-catenin expression, which ultimately suppressed the wingless-related integration site (Wnt) pathway, was also inferred from the Western blot analysis.

Numerous in vivo studies have been conducted to determine the anticarcinogenic potential of daidzein against osteosarcoma. Zhu and his colleagues [51] prepared an animal model by *s.c.* injecting pre-cultured 143B cells into male BALB/c nude mice. Daidzein treatment resulted in significant tumor volume reduction, due to direct inhibition of the Src-ERK axis. Wang et al. [51] also performed a similar kind of study by injecting 143B cells *s.c.* in male BALB/c nude mice to develop xenograft tumors and later on treating them with daidzein intraperitoneally at a dose of 20 mg/kg. Immunohistochemistry staining suggested reduced tumorigenicity via the downstream activation of the Wnt/β-catenin pathway.

### 3.11. Ovarian Cancer

Ovarian cancer, which is another deadly form of cancer, has caused more than approximately 200,000 deaths of women in the year 2022 globally [112]. Ranking eighth among the forms of cancer, with respect to its prevalence, it is mainly characterized by neoplastic growth on the epithelial surface of the ovaries, which later on metastasizes to the endometrium and other regions, like the peritoneal cavity, causing ascites [113,114,115,116,117].

Taylor et al. [118] conducted an in vitro study using cells obtained from either the ascites or the tumor of stage IIIC ovarian cancer patients. The cells were then cultured, and five different cell lines were prepared and renamed as UL-3C, UL-5, UL-6, UL-7, and UL-8. Daidzein at different concentrations (25–150 µM) significantly blocked the growth of the carcinoma cell lines, where UL-6 was found to be the most sensitive one, and UL-8 was the most resistant. Although no mechanistic pathway involved in this effect was evaluated, from the inferences, daidzein stood out as a potent chemotherapeutic isoflavone. Somjen et al. [53] cultured MLS human epithelial ovarian cancer cells, abundantly expressing ERβ mRNA. Treatment with daidzein inhibited cellular growth and imposed a cytotoxic effect in the cultured cells. A newer approach to potentiate the anti-ovarian cancer effect of daidzein was adopted by Chan et al. [119], where daidzein was treated with three distinct ovarian carcinoma cell lines, SKOV-3, A2780CP, and OVCAR-3. Antitumorigenic effect was concluded as cellular viability, proliferation, and metastasis were attenuated after daidzein treatment. Moreover, Western blot analysis supported the observed antitumor effects as expression of different markers was dampened, such as FAK and PI3K/Akt/glycogen synthase kinase (GSK) signaling leading to apoptotic death, along with p21/cyclin D1 leading to cell cycle arrest at S and G_2_/M phase. Additionally, expression of ERα was reduced in the treated ovarian cancer cells, as it has proto-oncogenic action, followed by overexpression of ERβ, which has potent anti-oncogenic activity.

Somjen et al. [53] conducted an in vivo experiment by inoculating MLS human ovarian epithelial carcinoma cells *s.c.* in female CD1 nude mice. Prominent minimization of tumor volume was observed following administration of daidzein, and fluorescence studies confirmed maximum uptake of the phytochemical by the tumor.

### 3.12. Pancreatic Cancer

Pancreatic cancer is considered one of the deadliest forms of carcinoma, prevalent in the gastrointestinal tract. Owing to poor diagnostic detectability, this variant of cancer currently has a less than five percent survival rate, causing worldwide mayhem in terms of mortality [120,121]. Pancreatic cancer can either be of exocrine or endocrine origin, mainly affecting glandular and squamous differentiation, whilst other forms may damage acinar differentiation [122,123,124].

Guo et al. [125] first evaluated the anti-pancreatic cancer activity of daidzein, using two different ER-positive pancreatic cancer cell lines, such as MiaPaCa-2 and PANC-1. Primarily, the antiproliferative effect of daidzein at various concentrations (0.1–100 μmol/L) was observed, which was later magnified microscopically for determining histological changes. Despite any reported mechanistic findings, only inferred results from the study indicated daidzein’s antineoplastic potency against carcinogenic cells, as cell growth and proliferation were aberrated. Supporting the previous report, Gundogdu et al. [89] experimented with different concentrations of daidzein on the MiaPaCa-2 cell line, and observed attenuation of cell viability and proliferation, establishing the phytochemical’s cytotoxicity. The aforementioned activity was measured using 2,3-bis-(2-methoxy-4-nitro-5-sulfophenyl)-2H-tetrazolium-5-carboxanilide (XTT) assay with daidzein possessing an IC_50_ value of 200 μM for 48 h. Additionally, the comet assay has confirmed the genotoxic potency of daidzein, as DNA tail length, tail moment, and tail intensity were all increased, hinting toward DNA damage in treated MiaPaCa-2 cells.

### 3.13. Prostate Cancer

Prostate carcinoma, a malignancy confined to the male population, is characterized by neoplastic growth in the prostate gland. The majority of prostate cancers are adenocarcinomas, where the origin is acinar, while the rest are of ductal origin [126,127,128]. The onset of neoplasm growth starts from the basal epithelial or the luminal cells located in the peripheral regions, which approximately comprise 70% of the total prostate tissue [129,130].

Kato et al. [54] made the first breakthrough in identifying the antineoplastic effect of daidzein in prostate cancer cells. Cell proliferation was determined by treating the PLS10 rat prostate cancer cell line with multiple concentrations of daidzein (25–100 μM) using water-soluble tetrazolium-1 and MTT assay. The conclusion from the observed results was highlighted on the antiproliferative potency of daidzein in a concentration-dependent manner. An extensive in vitro study on multiple prostate carcinoma cell lines, such as DU 145, PC-3, and LNCaP, was performed by Rabiau et al. [131] to validate the anticancer activity of daidzein with a proper mechanistic pathway establishment. Cell proliferation was attenuated, and cell cycle arrest at G_2_/M phase by daidzein at 110 μM concentration was the primary observation after the study. Several genes that were significantly downregulated consist of CDK7, glutathione S transferase E1 (GTSE1), karyopherin alpha 2 (KPNA2), murine Ki-67 (MKI67), and TP53. Moreover, angiogenesis of the cancer cells was also inhibited as several angiogenic markers were downregulated, such as epidermal growth factor (EGF) and insulin-like growth factor-1 (IGF-1), as determined via polymerase chain reaction (PCR) arrays.

Similar to the previous work, Gupta et al. [55] evaluated the in vitro anticancer potential of daidzein against PC-3 and C4-2B human prostate cancer cell lines. Aberration of cellular metastasis was the main antitumor effect exhibited by daidzein in a concentration-dependent manner. Western blot assay revealed milder cleavage of PARP, along with downregulated hypoxia-inducible factor (HIF)-α and apurinic/apyrimidinic endonuclease 1/redox factor-1 (APE/Ref-1) expressions, which were sharply upregulated due to tumorigenicity. Dong and colleagues (2013) concluded that daidzein was able to ameliorate prostate cancer growth by inducing apoptosis and aberrating cell proliferation when tested against LNCaP and C4-2B cell lines. Furthermore, cell cycle arrest at G_2_/M phase by 50 μM daidzein has also confirmed the antineoplastic activity of this phytochemical against prostate carcinoma [132]. A separate study reported daidzein’s inhibitory activity for prostate cancer cell invasiveness. Leiva et al. [133] demonstrated inhibitory activity of daidzein in terms of downregulated MMP-2 and MMP-9 gene expression within DU-145 and PC-3 cells that ultimately attenuated cell migration and invasiveness. As quantified by the RT-PCR method, daidzein at a concentration of 50 μM was able to exhibit such anticancer effects.

An in vivo research by Kato et al. [54] was focused on establishing anti-prostate carcinoma activity in animal models. This model was established through biweekly injection of 3,2′-dimethyl-4-aminobiphenyl (DMAB) *via s.c*. route to male F344 rats. Treatment with daidzein caused a significant reduction of ventral prostate neoplasms, followed by a reduction in the incidence of their recurrence. However, no signs of carcinoma reduction in the anterior prostate and seminal vesicles were visualized, concluding that the anti-prostate cancer efficacy of daidzein is only in the early stages of cancer and not in the late phases. Gupta et al. [55] also investigated the anti-prostate cancer effect of daidzein in vivo by preparing an animal model via inoculating PC-3/PI prostate tumor cells in the prostate gland of male BALB/c nu/nu nude mice. Regular treatment with daidzein 10.5 mg/kg body weight/day effectively inhibited tumor growth and progression, along with reducing the chances of newer neoplasm growth. Some additional inferences, such as dampening of HIF-α and APE/Ref-1 expression, also supported the antineoplastic activity of daidzein in prostate carcinoma.

### 3.14. Skin Cancer

Skin cancer, a malignancy with rising global incidence, is classified into melanoma and non-melanoma types. Melanoma most commonly arises from the irregular, abnormal proliferation of melanocytes, triggered by genetic anomalies and the activation of the inducible inflammatory pathway following carcinogen exposure [134,135,136,137,138].

Zhang et al. [139] found that daidzein had the potential to inhibit melanoma cells’ proliferation, invasion, and metastasis. For their study, K1735M2 murine melanoma cells were considered for their high metastatic activity, which were later treated with isoflavone daidzein at a concentration of 30 μmol/L. Significant attenuation of cellular migration and proliferation was inferred, as assayed by type I collagen-mediated melanoma cell invasion and MTT methods, respectively. Despite the adoption of a proper mechanistic pathway involved for the said oncotherapeutic effects, the inferred results established anticarcinogenic activity of daidzein against melanoma. Chang and Tsai [140] investigated the anti-melanogenic effect of daidzein against α-melanocyte-stimulating hormone-treated B16 melanoma cells. In their study, daidzein with a concentration of 50 μM reduced melanin concentration by dampening transcription of melanocortin 1 receptor (MC1R), phosphorylation of p38 MAPK, and expression of tyrosinase, tyrosinase-related protein-1 (TRP-1), and TRP-2. Additionally, daidzein stimulated phosphorylation of ERK1/2 and GSK-3β, which also contributed to exhibiting anti-melanogenic activity against B16 melanoma cells.

### 3.15. Concentration-Dependent Anticancer Activity: In Vitro Potency vs Physiological Relevance

Despite the variety of mechanisms of action of daidzein (Table 1), the concentrations required to achieve anticancer effects are found to be variable according to cancer type. The in vitro concentrations that are required to achieve anticancer effects are summarized in Table 3, categorized according to four concentration ranges. Analysis of these tables indicates that concentrations of 10–100 μM are generally required to exhibit anticancer effects, with effects being strongest above 50 μM, but potentially proliferative or ineffective at submicromolar concentrations.

**Table 1:** Anticancer effects and related mechanisms of action of daidzein based on in vitro experiments.

Cell Line Used	Concentrations/IC_50_ and Duration	Anticancer Effects	Mechanisms	References
*Brain cancer*				
LN229 (p53-mutant), NCH89 (human short-term glioblastoma culture)	50–200 μM, 24 h	↑Apoptosis	↓Bcl-2; ↑caspase-9	[61]
U251	50–100 μM, 48 h	↓Cell viability; ↑apoptosis	↑Bax; ↑caspase-3; ↓p-Akt; ↓p-mTOR	[63]
SH-SY5Y	100–1000 μg/mL, 24 h	↓Cell viability	↓PI3K; ↓Akt; ↑SOD; ↓GSH; ┴TBARS	[62]
*Breast cancer*				
MCF-7, MDA-MB-453	1–100 μM, 24–72 h	┴Proliferation; ↑apoptosis; ┴cell cycle at G_1_ and G_2_/M phase	↑p21^Cip1^; ↑p57^Kip2^; ↑caspase-9; ↓cyclin D; ↓CDK2; ↓CDK4; ↓CDK1; ↑accumulation of sub-G_0_ apoptotic fractions	[66]
MCF-7	25–100 μM, 24–72 h	↑Apoptosis, ↓cell viability	↑Bax; ↓Bcl-2; ↑cyt. c release; ↑caspase-7; ↑caspase-9; ↑ROS; ↓mitochondrial membrane potential;	[67]
MCF-7, SKBR-3, ZR-75-1	1–200 μM, 72 h	┴Proliferation; ↑apoptosis; ┴cellular growth	↓Erα; ↓ErbB2	[68]
MDA-MB-231	2.5–50 μM, 48 h	↓Cellular growth	↓MMP-2	[69]
MCF10DCIS.com	30 μM, 24 h	↓Cellular growth; ↓migration; ↓invasion	┴Gli1; ↓MMP-9	[70]
MCF-7, MDA-MB-231	3–10 μM, 48 h	┴Proliferation	Not reported	[39]
MCF-7, T47D	1–10 μM, 24 h	↑Apoptosis; ↓cell growth	↑ERα; ↓neuroglobin levels	[71]
MCF-7	25–100 μM, 24–72 h	┴Proliferation; ↑apoptosis	↑Bax; ↓Bcl-2; ↑caspase-3; ↑caspase-7; ↑ROS; ↑Erβ; ↓ERα	[72]
T47D	0.04 µg/mL, 48 h	↑Cytotoxicity	Not reported	[73]
MCF-7, SKBR-3, MDA-MB-231, T-47D	0.53 mM, 72 h	↑Cytotoxicity	Not reported	[74]
MDA-MB-231, MCF-7	25.4–33.2 µM, 48 h	↑Cytotoxicity; ↑apoptosis	↑lipid peroxidation; ↑ferroptosis; ↓GPX4; ↓FSP-1	[75]
*Cervical cancer*				
HeLa	6.25–100 µM, 24–96 h	┴Cellular growth; ↑apoptosis; ┴cell cycle at both G_0_/G_1_ and G_2_/M phases	↓hTERT	[77]
HeLa	0.54 µg/mL, 48 h	↑Cytotoxicity	Not reported	[73]
HeLa	20 μM, 24 h	↓Cellular growth; ↑apoptosis	↑ROS; ↑caspase-8; ↑caspase-9; ↓mitochondrial membrane potential	[78]
HeLa	20–40 μM, 48 h	↓Proliferation; ↑apoptosis	↑p21; ↑caspase-3; ↑PARP; ↓Bcl-xL; ↓MCM2; ↓PCNA; ┴cell cycle at G_1_ phase; ↓Cdc25C; ↓p-Cdk1; ↓cyclin B1; ↓mitochondrial membrane potential; ↓uPAR; ↓N-cadherin; ↓STAT3; ↓JNK	[79]
*Choriocarcinoma*				
JAR, JEG-3	10–30 µM, 24–72 h	↓Proliferation; ┴cell cycle at G_1_	↑p21; ↓c-Myc; ↓cyclin D1; ↓PCNA; ↓p-ERK	[41]
JAR, JEG-3	12.5–400 µM, 48 h	↓Viability; ↑apoptosis	↑Bax; ↓Bcl-2; ↑caspase-3; ↑caspase-9; ↑PARP	[84]
*Colon cancer*				
LoVo	0.1–100 μM, 48–120 h	┴Proliferation; ↑apoptosis; ┴cell cycle at G_0_/G_1_ phase	↑Caspase-3	[87]
HT-29	10–100 µM, 24–96 h	┴Proliferation	Not reported	[88]
HT-29	25–400 µM, 48 h	┴Proliferation; ↑apoptosis	↑FOXO3a; ↑caspase-8; ↓lipid droplet accumulation; ↓perilipin-1; ↓ADRP; ↓Tip-47; ↓vimentin; ↓PI3K	[89]
HT-29	200 µM, 48 h	↓Cell viability; ↑cytotoxicity	↑DNA damage; ↑DNA fragmentation	[90]
SW620	10–50 µM, 48 h	↓Proliferation	↓ERK; ↓Akt	[42]
*Gastric cancer*				
BGC-823	20–80 μM, 24–72 h	┴Proliferation; ↓cell viability; ↑apoptosis	↑Bax; ↓Bcl-2; ↑caspase-3; ↑PARP	[94]
BGC-823	29.90 μM, 24 h	↓Proliferation; ┴cell cycle at G_0_/G_1_ phase	↑ROS; ↓STAT3; ↓FAK; ↓Bcl-2; ↓cyclin D1; ↓MMP-2	[95]
*Liver cancer*				
SK-HEP-1	200–600 μM, 24 h	↓Cell viability	↑ROS; ↑probiotic lysate; ↑caspase-3; ↑caspase-7; ↑caspase-9; ↑cyt. c; ↓Bcl-2; ↓Bcl-xL; ↓Puma	[99]
BEL-7402	59.7 μM, 24 h	↑Apoptosis; ┴cell cycle at G_2_/M phase	↑ROS; ↑Bim; ↑caspase-7; ↓Bcl-2; ↓Bcl-xL; ↓Bid	[100]
HCCLM3, Hep3B	20–50 μM, 48 h	↓Cell viability; ┴Migration	↑Bax; ↑PARP; ↓Cdk1; ↓c-Myc; ↓Bcl-2; ↓survivin	[44]
*Lung cancer*				
A549, H1299	10–100 μM, 24 h	┴Proliferation; ↑apoptosis	↑STK4; ↑YAP-1; ↑caspase-3	[104]
A594, 95D	5–25 μM, 48 h	┴Proliferation	↓IL-6; ↓IL-8; ↓p65-NF-κB	[46]
H1299	10 μM, 6 h	↑Apoptosis	↑Caspase-9; ↑TP53	[105]
H1299	10 μM, 24 h	┴Proliferation	↑ENST00000608897.1; ↑ENST00000444196.1; ↓XR_242163.1; ↑ROPN1L; ↑FANCC; ↓TRHR; ↓IGF1	[106]
A549, H1975	100–300 μM, 24–48 h	┴Proliferation; ↑apoptosis; ┴cell cycle at G_0_/G_1_ phase	↑ASK1; ↑JNK; ↓EGFR; ↓STAT; ↓Akt; ↓ERK; ↑p53; ↓p27; ↓p21; ↓p16	[47]
LA795	1.39 μM, 24 h	↓Proliferation; ↓cell viability; ┴migration; ┴cell cycle at G_1_/S phase	↓TMEM16A	[48]
*Osteosarcoma*				
143B, U2OS	63.6–125 µM, 24 h	↓Proliferation; ┴cell cycle at G_0_/G_1_ phase	↓MAPK; ↓ERK; ↓Src	[51]
U2OS, HOS, MNNG/HOS, SJSA-1, 143B, MG63	10–160 μM, 24–72 h	↓Cell viability; ↓proliferation; ↑apoptosis; ┴metastasis	↓Wnt; ↓β-catenin	[52]
*Ovarian cancer*				
UL-3C, UL-5, UL-6, UL-7, UL-8	25–150 μM, 24–72 h	↑Cytotoxicity	Not reported	[118]
MLS	30–3000 nM, 48 h	↑Cytotoxicity	↑DNA damage	[53]
SKOV-3, A2780CP, OVCAR-3	10–50 μM, 48 h	↓Cell viability; ↓proliferation; ┴metastasis; ↑apoptosis; ┴cell cycle at G_2_/M phase	↓FAK; ↓PI3K; ↓Akt; ↓GSK; ↓p21; ↓cyclin D1; ↓ERα; ↑ERβ	[119]
*Pancreatic cancer*				
MiaPaCa-2, PANC-1	0.1–100 μM, 72 h	↓Cell viability	Not reported	[125]
MiaPaCa-2	200 µM, 48 h	↓Cell viability; ↑cytotoxicity	↑DNA damage; ↑DNA fragmentation	[90]
*Prostate cancer*				
PLS10	25–100 μM, 72 h	↓Cell viability; ↓proliferation	Not reported	[54]
DU 145, PC-3, LNCaP	110 μM, 48 h	↓Cell viability; ↓proliferation; ↓angiogenesis; ┴cell cycle at G_2_/M phase	↓CDK7; ↓GTSE1; ↓KPNA2; ↓MKI67; ↓TP53; ↓EGF; ↓IGF1	[131]
PC-3, C4-2B	15–100 μM, 72 h	┴Metastasis	↑PARP; ↓NF-κB; ↓HIF-α; ↓APE/Ref-1	[55]
LNCaP, C4-2B	50 μM, 48 h	┴Proliferation; ↑apoptosis; ┴cell cycle at G_2_/M phase	Not reported	[132]
DU-145, PC-3	50 μM, 24 h	↓Cell viability; ┴migration	↓MMP-2; ↓MMP-9	[133]
*Skin cancer*				
K1735M2	30 μmol/L, 72 h	↓Proliferation; ┴migration; ┴invasion	Not reported	[139]
B 16	50 μM	↓Proliferation	↓MC1R; ↓p38; ↓MAPK; ↓tyrosinase; ↓TRP-1; ↓TRP-2; ↑ERK1/2; ↑GSK-3β	[140]

The arrows indicate the decrease (↓) or increase (↑) and inhibition (┴). Abbreviations: ADRP, adipophilin/adipose differentiation-related protein; Akt, Ak stain transforming; APE/Ref1, apurinic/apyrimidinic endonuclease 1/redox factor-1; ASK1, apoptosis signal-regulating kinase 1; Bax, Bcl-2-associated X protein; Bcl-2, B-cell lymphoma 2; Bcl-xL, B-cell lymphoma-extra-large; BCRP, breast cancer resistance protein; Bid, BH3-interacting domain death agonist; Bim, Bcl-2 interacting mediator of cell death; Cdc25C, cell division cycle 25C; CDK, cyclin-dependent kinase; Cdk1, cyclin-dependent kinase 1; CDK7, cyclin-dependent kinase 7; c-Myc, myelocytomatosis oncogene; cyt. c, cytochrome c; DNA, deoxyribonucleic acid; EGF, epidermal growth factor; EGFR, epidermal growth factor receptor; EMT, epithelial–mesenchymal transition; ErbB2, epidermal growth factor receptor 2; ER, estrogen receptor; ERα, estrogen receptor alpha; ERβ, estrogen receptor β; ERK, extracellular signal-regulated kinase; FAK, focal adhesion kinase; FANCC, Fanconi Anemia complementation group C; FOXO3a, Forkhead box O 3a; FSP-1, ferroptosis suppressor protein 1; Gli1, glioma-associated oncogene homolog 1; GPX4, glutathione peroxidase 4; GSH, reduced glutathione; GSK, glycogen synthase kinase; GSK-3β, glycogen synthase kinase-3β; GTSE1, glutathione S transferase E1; HIF-α, hypoxia-inducible factor-α; hTERT, human telomerase reverse transcriptase; IGF1, insulin-like growth factor 1; IL-6, interleukin-6; IL-8, interleukin-8; JNK, c-Jun N-terminal kinase; KPNA2, karyopherin alpha 2; MAPK, mitogen-activated protein kinase; MC1R, melanocortin 1 receptor; MCM, minichromosome maintenance; MKI67, murine Ki-67; MMP-2, matrix metalloproteinase-2; MMP-9, matrix metalloproteinase-9; mTOR, mammalian target of rapamycin; NF-κB, nuclear factor-κB; PARP, poly (ADP-ribose) polymerase; PCNA, proliferating cell nuclear antigen; PI3K, phosphoinositide 3-kinase; Puma, p53 upregulated modulator of apoptosis; ROPN1L, rhophilin-associated tail protein 1 like; ROS, reactive oxygen species; SOD, superoxide dismutase; STAT3, signal transducer and activator of transcription 3; STK4, serine/threonine-protein kinase 4; TBARS, thiobarbituric acid-reactive substances; TIMP, tissue inhibitors of metalloprotease; Tip-47, tail-interacting protein 47; TMEM16A, transmembrane protein 16A; TNF-α, tumor necrosis factor-α; Topo I, topoisomerase I; TP53, tumor protein 53; TRHR, thyrotropin-releasing hormone receptor; TRP-1, tyrosinase-related protein-1; TRP-2, tyrosinase-related protein-2; uPAR, urokinase plasminogen activator receptor; Wnt, wingless-related integration site; YAP-1, yes-associated protein-1.

**Table 2:** Anticancer effects and related mechanisms of action of daidzein based on in vivo experiments.

Animal Tumor Models	Anticancer Effects	Mechanisms	Dose (Route)	Duration	References
*Breast cancer*					
DMBA-induced mammary tumors in female Sprague Dawley rats	↓Tumor development; ↑apoptosis	Not reported	0.69–6.9 mg/kg/day (oral)	3 weeks	[40]
MCF-7 cell xenograft into female athymic nude mice		↑Caspase-3	6.9 mg/kg/day (oral)	4 weeks	
Female C57BL/6J mice	↓Tumor growth	↓BRF1; ↓BRF2	6.9 mg/kg/day (oral)	3 weeks	[39]
*Choriocarcinoma*					
JEG-3 cells xenograft in male BALB/c nude mice	┴Tumor growth	↓c-Myc; ↓cyclin D1; ↓PCNA; ↓p-ERK	10 mg/kg/day (oral)	4 weeks	[41]
*Colon cancer*					
DMH/DSS male rat model	↓Tumor progression	↑DNA damage; ↓AREG; ↓CXCL1; ↓MMP-9	5 and 10 mg/kg, three times per week (oral)	8 weeks	[42]
*Head and neck cancers*					
Hypopharyngeal SCC xenograft in mice	↓Tumor growth	↑PPARγ; ↓ANGPT4/Tie2; ↓TGF-β; ↓IL-35; ↓Ki-67	20–40 mg/kg (i.p.)	Not specified	[43]
*Liver cancer*					
Hep3B cells xenograft in female nude mice	┴Tumor growth	↓TPI1	50 and 100 mg/kg three times per week (i.p.)	24 days	[44]
DENA/CCl_4_-induced hepatocellular cancer in male adult Wistar rats	↓Tumor progression	↓ALP; ↓ALT; ↓AST; ↓AFP; ↓GPC-3; ↓VEGF; ↓IL-6; ↓TNF-α; ↓CRP	20 and 40 mg/kg/day (oral)	12 weeks	[45]
*Lung cancer*					
B16F-10 cells xenograft in male C57BL/6 mice	↓Tumor nodules	↓Hydroxyproline content in lungs	200 μmol/kg on every alternate 10 days (oral)	3 weeks	[49]
A594 cells xenografted in male BALB/c nude mice	┴Tumorigenesis	↓p65-NF-κB; ↓Ki-67	20 mg/kg/day (i.p.)	3 weeks	[46]
Male nu/nu nude mice bearing A549 xenograft model	┴Tumor growth	↑ASK1; ↑JNK; ↓EGFR	300 mg/kg twice a week (oral)	5 weeks	[47]
Benzo(a)pyrene-induced lung cancer in male Swiss albino mice	┴Tumorigenesis	↓IgA; ↑IgG; ↑IgM; ↓AHH; ↓ADA; ↓LDH; ↓GGT; ↓IL-1β; ↓IL-6; ↓TNF-α; ↓PCNA; ↓CYP1A1; ↓Nrf2	20 mg/kg/day (oral)	6 weeks	[50]
LA795 cells xenograft in female BALB/c mice	┴Tumor growth	Not reported	1.25, 2.5 and 5 mg/kg for every 3 days (i.p.)	4 weeks	[48]
*Osteosarcoma*					
Male BALB/c nude mice bearing 143B xenograft model	↓Tumor volume	↓ERK; ↓Src	20 mg/kg every 2 days (i.v.)	16 days	[51]
Male BALB/c nude mice bearing 143B xenograft model	↓Tumor volume; ↓cell viability; ↓metastasis	↓Wnt; ↓β-catenin	20 mg/kg/day (i.p.)	27 days	[52]
*Ovarian cancer*					
MLS cells xenograft in female CD1 nude mice	↓Tumor volume; ↓tumor progression	↑DNA damage	8 mg/kg/day (oral)	17 days	[53]
*Prostate cancer*					
DMAB-induced prostate cancer in male F344 rats	┴Tumorigenesis	↑Testosterone level; ↑estradiol	0.1% with diet for every 3 weeks (oral)	60 weeks	[54]
			0.1% with diet/day (oral)	4 weeks	
Male BALB/c nu/nu nude mice bearing PC-3/PI xenograft model	↓Tumor growth	↓HIF-α; ↓NF-κB	10.5 mg/kg/day (oral)	36 days	[55]

The arrows indicate the decrease (↓) or increase (↑) and inhibition (┴). Abbreviations: ADA, adenosine deaminase; AFP, α-fetoprotein; AHH, aryl hydrocarbon hydroxylase; ALP, alkaline phosphatase; ALT, alanine aminotransferase; ANGPT4, angiopoietin 4; Apaf-1, apoptotic protease-activating factor 1; AREG, amphiregulin; ASC, apoptosis-associated speck-like protein; ASK1, apoptosis signal-regulating kinase 1; AST, aspartate aminotransferase; Bax, Bcl-2-associated X protein; Bcl-xL, B-cell lymphoma-extra-large; BRF, butyrate response factor; Cav-1, caveolin-1; CDK2, cyclin-dependent kinase 2; CDK4, cyclin-dependent kinase 4; c-Myc, myelocytomatosis oncogene; CRP, C-reactive protein; CXCL1, chemokine ligand; CYP1A1, cytochrome P450, family 1, subfamily A, polypeptide 1; DMAB, 3,2′-dimethyl-4-aminobiphenyl; DMBA, 7,12-dimethylbenz[a]anthracene; DMH, 1,2-dimethylhydrazine; DNA, deoxyribonucleic acid; DSS, dextran sulfate sodium; EGFR, epidermal growth factor receptor; ERK, extracellular signal-regulated kinase; FADD, Fas-associated protein with death domain; GGT, γ-glutamyl transferase; GPC-3, glypican-3; HIF-α, hypoxia-inducible factor-α; HIF-1α, hypoxia-inducible factor-1α; IgA, immunoglobulin-A; IgG, immunoglobulin-G; IgM, immunoglobulin-M; IL, interleukin; IL-1β, interleukin-1β; IL-6, interleukin-6; JNK, c-Jun N-terminal kinase; LDH, lactate dehydrogenase; MDA, malondialdehyde; MMP-9, matrix metalloproteinase-9; NF-κB, nuclear factor-κB; Nrf2, nuclear factor erythroid 2-related factor 2; PCNA, proliferating cell nuclear antigen; p-ERK, phosphorylated extracellular signal-regulated kinase; PPARγ, peroxisome proliferator-activated receptor γ; SOD, superoxide dismutase; TGF-β, transforming growth factor-β; Tie2, tyrosine kinase with immunoglobulin-like and EGF-like domains 2; TNF-α, tumor necrosis factor-α; VEGF, vascular endothelial growth factor; Wnt, wingless-related integration site.

**Table 3:** Summary of effective in vitro concentrations of daidzein and their association with anticancer effects across cancer types.

Concentration Range	Cancer Types Showing Anticancer Effects	Representative Cell Lines & Key Effects	Biphasic or Weak Activity	References
≤1 μM	Limited or none (occasionally proliferative)	LoVo (colon): proliferation at 0.1–1 μM; MCF-7 (breast): proliferation at 1 μM	Biphasic response common; weak or stimulatory in ER+ models	[66,68,87]
1–10 μM	Breast, Cervical (low end), Lung (low end), Prostate (low end)	MCF-7 & MDA-MB-231 (breast): growth inhibition, apoptosis & invasion suppression at 3–10 μM; HeLa (cervical) at lower end; A549/H1299 (lung) at 5–10 μM	Emerging antiproliferative and anti-invasive activity; ferroptosis initiation in TNBC near the upper end	[39,46,47,66,67,68,69,70,77,133]
10–50 μM	Breast, Cervical, Choriocarcinoma, Colon, Gastric, Lung, Ovarian, Pancreatic, Prostate, Skin	MCF-7 (breast) IC_50_ ~50 μM, apoptosis; HeLa (cervical) 20 μM, G1 arrest & apoptosis; JAR/JEG-3 (choriocarcinoma) 10–30 μM, G1 arrest; HT-29/LoVo (colon) 10–50 μM, apoptosis; BGC-823 (gastric) ~30 μM; A549/H1299 (lung) 10–50 μM; DU145/PC-3 (prostate) 50 μM; K1735M2 (skin) 30 μM	Most consistent range for antiproliferation, cell cycle arrest (G_0_/G_1_ or G_2_/M), apoptosis, and reduced migration/invasion	[41,42,46,47,53,54,55,66,67,72,77,78,79,84,87,88,89,90,94,95,104,105,106,118,119,125,131,132,133,139]
>50 μM	Brain (glioma), Liver, Osteosarcoma, some Breast/Lung/Prostate	LN229/U251 (glioma) 50–200 μM, intrinsic apoptosis; SK-HEP-1/BEL-7402 (liver) 59–600 μM, ROS & caspase activation; 143B/U2OS (osteosarcoma) 63–125 μM, G0/G1 arrest & Wnt inhibition; high doses in breast (100–200 μM) and lung models	Strongest effects on viability, ROS generation, mitochondrial damage, and apoptosis; often required for glioma, liver, and osteosarcoma	[44,47,51,52,54,55,61,62,63,66,67,68,99,100,104,131,132]

Abbreviations: ER, estrogen receptor; ROS, reactive oxygen species; TNBC, triple-negative breast cancer.

## 4. Anticancer Potential of Daidzein in Combination with Other Therapeutic Interventions

From various studies emphasizing the antineoplastic activity of daidzein against various forms of cancers, it was found that in many studies, daidzein was able to exhibit more potent action when administered with another natural compound or with other chemotherapeutic drugs. Significant improvement in anticancer effects includes minimization of dosage requirements, preventing toxicity, and improving the therapeutic potency of daidzein. The following subsections focus on the anticancer potential of daidzein in combination with either natural agents or with approved anticancer drugs.

### 4.1. Daidzein in Combination with Natural Agents

#### 4.1.1. Breast Cancer

A recent study explored the presence of daidzein and genistein in legume sprouts and their combined activity against the MCF-7 cell line. The sprouts of *Glycine max* (soybean), *Macrotyloma uniflorum* (horse gram), and *Cicer arietinum* (chickpea) had the maximum levels of these isoflavones, and genistein was present in higher amounts than daidzein. Incubation of the MCF-7 cell line with isoflavone extracts for 24 h inhibited the growth of cells in a concentration-dependent manner, and their IC_50_ values were 150.9, 266.36, and 379.55 µg/mL for *G. max*, *M. uniflorum*, and *C. arietinum*, respectively (Table 4). Cellular growth and proliferation were aberrated with higher concentrations of the plant extracts, thereby eliciting potent antineoplastic activity. Irrespective of promising preclinical results, no mechanistic activity was reported [141].

An in vivo pharmacological combination of daidzein-conjugated methionine γ-lyase (C115H-Dz) and propiin, a natural organosulfur compound, was tested in female BALB/c nude mice bearing SKBR-3 xenografts (Table 5). In this study, treatment caused an 85% inhibition of tumor growth, demonstrating the therapeutic potential of daidzein-based targeting approaches in the treatment of breast cancer. The lack of mechanistic results formed a major limitation for this study [74].

#### 4.1.2. Gastric Cancer

Ge et al. [95] concluded a study to emphasize the combinatorial effect of daidzein with another isoflavonoid compound, puerarin, against BGC-823 gastric cancer cell lines. Cellular proliferation was minimized by the combination at a concentration of 19.64 ± 0.71 μM (IC_50_), which was much less than that of the individual isoflavones. Moreover, enhanced ROS generation influenced the cell death process, along with cell cycle arrest at the G_0_/G_1_ phase with increased apoptotic death. Moreover, suppression of STAT3 and FAK phosphorylation directly suppressed and inhibited the expression of cyclin D1, Bcl-2, and MMP-2, which are crucial survival markers of gastric carcinoma.

#### 4.1.3. Liver Cancer

The first combinatorial approach for treating hepatic cancer was performed by Zakaria et al. [142], in which daidzein, along with chicory extract, was administered as a therapeutic intervention against DENA-induced hepatocellular cancer in male albino rats. Daidzein with chicory extract significantly downregulated the genetic expression of HIF-1α and caveolin-1 (Cav-1), along with complete inhibition of VEGF and MMP-9. Moreover, oxidative stress marker malondialdehyde (MDA) was reduced, and SOD was increased, supporting oxidative cell damage leading to cell death. Later, Abdel-Hamid et al. [143] demonstrated the anti-hepatic cancer activity of daidzein against DENA-induced hepatocellular carcinoma in adult male Wistar albino rats. Cell cycle arrest at G_1_/S and S/G_2_ phases was observed in the experimental animals after treatment with daidzein. Minimizing the expression of the cyclin D1/CDK4 axis, as well as the cyclin A/CDK2 axis, was the mechanistic result that led to the establishment of the antitumor potential. The dampened cellular proliferation due to inhibitory effect on Ki-67 expression and stimulated apoptotic cell death by downregulating Bcl-2 expression contributed to the anti-hepatocellular carcinoma effect.

#### 4.1.4. Prostate Cancer

Dong and colleagues [132] conducted research that demonstrated the synergistic effect of daidzein with genistein against LNCaP and C4-2B cell lines. Both daidzein and genistein were used at a concentration varying from 25 μM to 50 μM for 48 h. The main observations from the study included amelioration of prostate cancer growth by inducing apoptosis and aberrant cell proliferation. Furthermore, cell cycle arrest at G_2_/M phase from the combination was also inferred and posed a similar kind of cytotoxic effect as compared to daidzein alone. Together, both phytochemicals contributed to a synergistic effect that improved the antineoplastic effect against prostate carcinoma [132].

**Table 4:** In vitro studies on the combined effects of daidzein with natural agents.

Compound Tested	Cell Line Used	Concentrations/ IC_50_/CI_50_	Duration	Anticancer Effects	Mechanisms	References
*Breast cancer*						
Daidzein + genistein	MCF-7	Not reported	24 h	┴Cell growth; ↓proliferation	Not reported	[141]
*Gastric cancer*						
Daidzein + puerarin	BGC-823	19.64 ± 0.71µM	24 h	↓Proliferation; ┴cell cycle at G_0_/G_1_ phase	↑ROS; ↓STAT3; ↓FAK; ↓Bcl-2; ↓cyclin D1; ↓MMP-2	[95]
*Prostate cancer*						
Daidzein + genistein	LNCaP, C4-2B	50 μM	48 h	┴Proliferation; ↑apoptosis; ┴cell cycle at G_2_/M phase	Not reported	[132]

The arrows indicate the decrease (↓) or increase (↑) and inhibition (┴). Abbreviations: Bcl-2, B-cell lymphoma 2; ER, estrogen receptor; FAK, focal adhesion kinase; MMP-2, matrix metalloproteinase-2; ROS, reactive oxygen species; STAT3, signal transducer and activator of transcription 3.

**Table 5:** In vivo studies on the combined effects of daidzein with natural agents.

Material Tested	Animal Tumor Models	Anticancer Effects	Mechanisms	Dose	Duration	References
*Breast cancer*						
Daidzein-conjugated methionine γ-lyase + propiin	Female BALB/c nude mice bearing SKBR-3 xenograft model	┴Tumor growth	Not reported	30 U (Daidzein-conjugated methionine γ-lyase in 200 mL potassium phosphate buffer) (i.p.) + 3 mg (propiin) (intratumorally)	10 d	[74]
*Liver cancer*						
Daidzein + chicory extract	DENA-induced hepatocellular tumor in male albino rats	↓Tumor development	↑SOD; ↓HIF-1α; ↓Cav-1; ↓VEGF; ↓MMP-9; ↓MDA	20 mg/kg/day (i.p.)	4 weeks	[142]
	DENA-induced hepatocellular tumor in adult male Wistar albino rats	┴Tumor growth; ↑apoptosis; ┴cell cycle at G_1_/S phase; ┴cell cycle at S/G_2_ phase	↓Cyclin D; ↓CDK4; ↓cyclin A; ↓CDK2	20 mg/kg/day (i.p.)	4 weeks	[143]

The arrows indicate the decrease (↓) or increase (↑) and inhibition (┴). Abbreviations: Apaf-1, apoptotic protease-activating factor 1; Bax, Bcl-2-associated X protein; Bcl-xL, B-cell lymphoma-extra-large; Cav-1, caveolin-1; CDK2, cyclin-dependent kinase 2; CDK4, cyclin-dependent kinase 4; DENA, diethylnitrosamine; FADD, Fas-associated protein with death domain; HIF-1α, hypoxia-inducible factor-1α; IL-6, interleukin-6; MDA, malondialdehyde; MMP-9, matrix metalloproteinase-9; SOD, superoxide dismutase; VEGF, vascular endothelial growth factor.

### 4.2. Daidzein in Combination with Other Chemotherapeutic Agents

#### 4.2.1. Breast Cancer

A study investigated the synergistic potential of the combination of daidzein with centchroman, a selective ER modulator, in breast cancer cells. Exposure of MCF-7 and MDA-MB-231 cell lines to daidzein (50 μM) and centchroman (10 μM), either alone or in combination, at the same concentration, for 24–48 h showed that the combination was more cytotoxic than monotherapy, while being less harmful to non-tumorigenic human mammary epithelial MCF-10A cells (Table 6). Combination index (CI) analysis also showed synergism between daidzein and centchroman. Mechanistically, the combination treatment caused apoptosis, as indicated by Annexin V/PI staining, terminal deoxynucleotidyl transferase dUTP nick-end labeling (TUNEL) assay, and caspase activation, along with mitochondrial membrane potential disruption and increased ROS production. Downregulation of survival signaling proteins PI3K, Akt, and mTOR was further detected by Western blotting, indicating inhibition of the PI3K/Akt/mTOR pathway as a major mechanism for the synergistic effects observed [144]. These findings suggest the possible involvement of the PI3K/Akt/mTOR axis, although further studies are required to confirm these findings. Guo et al. [145] examined the synergistic actions of daidzein combined with topotecan, a chemotherapeutic drug, against two breast cancer cell lines. In MCF-7 and drug-resistant MCF-7/ADR cell lines, exposure to daidzein (5–50 μM, 24–72 h) in combination with topotecan (0.5–5 μM) elicited a significant synergistic antiproliferative effect, with a CI of 0.10–0.66. Mechanistically, daidzein augmented topotecan-mediated topoisomerase I inhibition in the MCF-7 cell line, leading to a G_2_/M phase accumulation and increased apoptotic cell death. In addition, in the case of MCF-7/ADR cell line, daidzein overcame topotecan resistance through ERα and breast cancer resistance protein (BCRP) transporter downregulation, leading to increased intracellular drug levels and restored topotecan sensitivity.

In vivo assessment of the combinatorial effect was demonstrated by feeding female Sprague Dawley rats with daidzein-supplemented diets (105 mg/kg diet) alone or combined with tamoxifen (0.125 mg/kg diet). One week later, the tumors were induced by a single intragastric dose of DMBA. The tamoxifen–daidzein diet decreased tumor multiplicity by 76%, incidence by 35%, and tumor burden by over 95%, in addition to advancing tumor latency by 62% over controls (Table 7). Additionally, oxidative DNA damage, as indicated by 8-oxo-deoxyguanosine levels in mammary glands, was highly diminished in the tamoxifen–daidzein group. This research showed that daidzein enhances the breast cancer chemopreventive activity of tamoxifen in vivo [146]. Later, the anticancer activity of daidzein in combination with topotecan, a cytotoxic topoisomerase I inhibitor, was also confirmed in xenograft models of MCF-7 and MCF-7/ADR cells. Mice with MCF-7 xenograft tumors were administered topotecan (3 mg/kg, *i.p*.) in combination with daidzein (5 mg/kg, oral) for 15 days, while mice with MCF-7/ADR xenografts were administered topotecan (1 mg/kg, *i.p*.) in combination with daidzein (5 mg/kg, oral) for the same period. Combination therapy significantly inhibited tumor growth in comparison to topotecan monotherapy and exerted stronger inhibition on drug-sensitive and drug-resistant models. These results show that daidzein not only synergizes with topotecan’s antitumor activity but also effectively overcomes BCRP-mediated drug resistance in vivo [145].

#### 4.2.2. Colon Cancer

Altinkaynak et al. [147] investigated daidzein and its combination with 5-fluorouracil (5-FU), an antimetabolite causing thymidylate synthase inhibition and halting DNA/RNA synthesis, against the Caco-2 colorectal adenocarcinoma cell line. The cells were treated with daidzein at 0.2–1.6 μM for 24–72 h, and viability was evaluated using trypan blue and MTT assays. In combination experiments, the cell line treated with daidzein (1–4 μM) along with 5-FU (50 μM) presented synergistic cytotoxicity. Mechanistically, enzyme-linked immunosorbent assay (ELISA) showed enhanced release of substance P, while in silico docking showed robust binding of daidzein with apoptosis-related receptors TNF-related apoptosis-inducing ligand receptor (TRAILR)-2, TNFαR-1, and interferon-γ receptor (IFNγR)-1 over 5-FU. The findings indicate that daidzein is associated with apoptosis in Caco-2 cells through TRAILR-2/TNFαR-1/IFNγR-1 receptor-mediated pathways and potentiates the efficacy of 5-FU.

#### 4.2.3. Lung Cancer

Mhone et al. [47] found that daidzein with combination of gefitinib, an approved chemotherapeutic drug for lung carcinoma, inhibited A549 and H1975 cell proliferation and induced apoptosis by directly inducing c-Jun nuclear translocation through ROS/ASK1/JNK and dampening EGFR-STAT/Akt/ERK pathways. The concentration combination that exhibited maximum potency was 10 μM of gefitinib with 300 μM of daidzein. Additionally, modifications in cell cycle regulators, such as upregulation of p53, followed by marked downregulation of p27, p21, and p16, caused abrupt cell cycle arrest at G_0_/G_1_ phase, which indicated the synergistic effect of daidzein with gefitinib.

Similar observations from in vivo studies were also inferred. Daidzein in combination with gefitinib caused apoptotic cell death in the A549 xenografted tumor in male nu/nu nude mice, as reported by Mhone et al. [47]. Reported mechanistic results were upregulation of ASK-1/JNK pathway, along with cleaving PARP-1 and caspase-3, as confirmed by Western blot analysis. The combined therapy was able to ameliorate tumor growth more effectively than monotherapy.

#### 4.2.4. Oral Squamous Cell Cancer

Ranking 15th among the forms of cancer-associated deaths, oral squamous cell cancer is a heterogeneous malignant neoplasm associated with various pathways for its pathogenesis [148]. Oral microbiome modulations, genetic predisposition, and immunomodulatory changes are considered to be some of the important factors for the growth of this neoplasm [149]. Excessive use of carcinogenic materials, including smoking and drinking, also plays a huge role in the epidemiology of this ailment. Growth of some viruses, such as human papillomavirus (HPV) and Epstein–Barr virus (EBV), in the buccal cavity can also lead to the pathogenesis of oral squamous cell carcinoma [150,151].

Only one recent study by Kang et al. [152] has been performed that helped to gain insight into the activity of daidzein in combination with cisplatin, a platinum-based cytotoxic drug causing the formation of cross-linking DNA and halting cell division, against oral squamous cell carcinoma and elucidate the molecular mechanism involved. Ca9-22 human gingival carcinoma cell lines were cultured and subjected to an assay for the in vitro analysis. Notable reduction of cell migration with aberrated proliferation, viability, and metastasis were the main inferences from the study. Modulation of various markers, such as dampening the expression of MMP-2 and MMP-9, complete inactivation of the MAPK pathway and its markers, such as phosphorylated ERK1/2 and phosphorylated p38, along with downregulation of epithelial–mesenchymal transition (EMT) in the carcinoma cells, were the prime mechanistic pathways involved for providing the antineoplastic effect of the combination treatment.

#### 4.2.5. Osteosarcoma

Wang et al. [52] investigated the action of daidzein in minimizing chemoresistance of cisplatin toward several osteosarcoma cell lines, namely SJSA-1 and 143B cells. The anticancer effects were comparable to those of daidzein alone, with a marked reduction in cellular migration and invasion identified as the primary mechanistic pathway modulations. As a direct effect of the minimization of chemoresistance, the recorded IC_50_ of the combination was significantly lower than the individual concentrations. Additionally, downregulation of β-catenin expression, which ultimately suppressed the Wnt pathway, was also inferred from the Western blot analysis.

Supporting the above results, Wang et al. [52] further investigated the synergistic effect of daidzein with chemotherapeutic drug cisplatin by injecting 143B cells in male BALB/c nude mice and later on treating them with the combination therapy. Primary inferences were attenuation of tumor weight and volume, which signified antineoplastic effect, and from immunohistochemistry staining, it was concluded that daidzein improved chemosensitivity in osteosarcoma tumors by reducing tumorigenicity through downregulation of the Wnt/β-catenin pathway.

**Table 6:** In vitro anticancer studies of daidzein in combination with chemotherapeutic drugs.

Compound Tested	Cell Line Used	Concentrations/IC_50_ and Duration	Duration	Anticancer Effects	Mechanisms	References
*Bone cancer*						
Daidzein + cisplatin	SJSA-1, 143B	40 μM (daidzein) + 1 μM (cisplatin)	24–72 h	┴Migration; ┴invasion	↓Wnt; ↓β-catenin	[52]
*Breast cancer*						
Daidzein + centchroman	MCF-7 + MDA-MB-231	50 μM (daidzein) + 10 μM (centchroman)	24–48 h	↑Apoptosis	↑ROS; ↓mitochondrial membrane potential; ↓PI3K; ↓Akt; ↓mTOR	[144]
Daidzein + topotecan	MCF-7, drug-resistant MCF-7/ADR	5–50 μM (daidzein) + 0.5–5 μM (topotecan)	24–72 h	↓Proliferation; ↑apoptosis	┴Topo I; ↓ERα; ↓BCRP transporter	[145]
*Colon cancer*						
Daidzein + 5-fluorouracil	Caco-2	1–4 μM (daidzein) + 50 μM (5-FU)	24–72 h	↓Proliferation	Not reported	[147]
*Lung cancer*						
Daidzein + gefitinib	A549, H1975	300 μM (daidzein) + 10 μM (gefitinib)	24–48 h	┴Proliferation; ↑apoptosis; ┴cell cycle at G_0_/G_1_ phase	↑ASK1; ↑JNK; ↓EGFR; ↓STAT; ↓Akt; ↓ERK; ↑p53; ↓p27; ↓p21; ↓p16	[47]
*Oral squamous cell cancer*						
Daidzein + cisplatin	Ca9-22	100 μM (daidzein) + 12.5 μM (cisplatin)	24 h	┴Migration; ↓cell viability; ↓proliferation; ┴metastasis	↓MMP-2; ↓MMP-9; ↓MAPK; ↓ERK1/2; ↓p38; ↓EMT	[152]

The arrows indicate the decrease (↓) or increase (↑) and inhibition (┴). Abbreviations: Akt, Ak stain transforming; ASK1, apoptosis signal-regulating kinase 1; BCRP, breast cancer resistance protein; EGFR, epidermal growth factor receptor; EMT, epithelial–mesenchymal transition; ERα, estrogen receptor α; ERK, extracellular signal-regulated kinase; JNK, c-Jun N-terminal kinase; MAPK, mitogen-activated protein kinase; MMP-2, matrix metalloproteinase-2; MMP-9, matrix metalloproteinase-9; mTOR, mammalian target of rapamycin; PI3K, phosphoinositide 3-kinase; ROS, reactive oxygen species; STAT, signal transducer and activator of transcription; Topo I, topoisomerase I; Wnt, wingless-related integration site.

**Table 7:** In vivo anticancer studies of daidzein in combination with chemotherapeutic drugs.

Material Tested	Animal Tumor Models	Anticancer Effects	Mechanisms	Dose	Duration	References
*Bone cancer*						
Daidzein + cisplatin	Male BALB/c nude mice bearing 143B xenograft model	↓Tumor volume; ↓cell viability; ↓metastasis	↓Wnt; ↓β-catenin	20 mg/kg (daidzein) + 5 mg/kg (cisplatin)	27 days	[52]
*Breast cancer*						
Daidzein + tamoxifen	Female Sprague Dawley rats	↓Tumor growth	↓DNA damage	105 mg/kg (daidzein) + 0.125 mg/kg (tamoxifen)	4 weeks	[146]
Daidzein + topotecan	MCF-7 xenografts in female nude mice	↓Tumor growth	Not reported	5 mg/kg (daidzein) + 3 mg/kg (topotecan)	15 days	[145]
	MCF-7/ADR xenografts in female nude mice			5 mg/kg (daidzein) + 1 mg/kg (topotecan)		
*Lung cancer*						
Daidzein + gefitinib	Male nu/nu nude mice bearing A549 xenograft model	┴Tumor growth	↑ASK1; ↑JNK; ↓EGFR	300 mg/kg (daidzein) + 100 mg/kg (gefitinib)	5 weeks	[47]

The arrows indicate the decrease (↓) or increase (↑) and inhibition (┴). Abbreviations: ASK1, apoptosis signal-regulating kinase 1; DNA, deoxyribonucleic acid; EGFR, epidermal growth factor receptor; JNK, c-Jun N-terminal kinase; Wnt, wingless-related integration site.

### 4.3. Nanoformulations of Daidzein Exhibiting Anticancer Activities

Nanoformulations or nanomedicine are very crucial for developing precise treatments as well as modulating the release of drugs from their respective formulations. For cancers also, drug nanocarriers provide an immense therapeutic advantage for improving phytochemicals’ circulation through the systemic route, as well as potentiate their therapeutic index [153,154,155,156,157]. Simultaneously, solubility, stability, absorption, and half-life of phytocompounds are increased, with minimized fast-pass metabolism rate and toxicity of the phytocompound toward normal cells [158]. The big lacuna of using phytocompounds for cancer therapy is constrained by numerous physicochemical and pharmacokinetic parameters, including poor bioavailability, water solubility, and selectivity toward the respective site of action. These drawbacks are overcome by using phytochemicals dispensed in the form of nanomedicines [159,160,161,162,163]. In the last decade, various nanocarriers, such as microcapsules, nanosuspensions, and solid lipid nanoparticles, have been investigated to enhance the anticancer activity, stability, and bioavailability of daidzein. In the following subsections, we present several studies that have been performed involving the application of different types of nanoformulations of daidzein against various forms of cancer.

Table 8 summarizes the key characteristics, anticancer effects, and mechanisms of action of all daidzein-based nanoformulations investigated to date in in vitro cancer models. This table allows for direct comparison of formulation types, cell line specificity, and therapeutic efficacy.

#### 4.3.1. Colon Cancer

Sanatkar et al. [164] formulated a new chitosan-encapsulated daidzein (CED) to enhance delivery and anticancer activity in colon cancer. The microcapsules (460 nm diameter, 64.3% entrapment efficiency, +34 mV zeta potential) possessed a sustained release profile, with ~38% release in simulated gastric/intestinal fluid and ~79% release in simulated colonic fluid within 24 h. CED treatment (25–100 µg/mL, 48 h) strongly suppressed the proliferation of the HT-29 colorectal cancer cell line. Mechanistically, CED triggered apoptosis, as indicated by elevated caspase-3 expression and elevated apoptotic fraction. These findings suggest that encapsulation may enhance cellular uptake and anticancer activity of daidzein in vitro; however, its impact on systemic bioavailability remains to be validated in vivo. Becit-Kizilkaya et al. [165] explored the anticancer activity of a daidzein nanosuspension (DZ-NS) (233 nm particle size, 0.29 polydispersity index) in Caco-2 colorectal cancer cells. After 24 h exposure, DZ-NS (IC_50_: 35.34 μM) was significantly more cytotoxic than free daidzein (IC_50_: 240.3 μM). The mechanistic studies showed that treatment with DZ-NS increased p53 but decreased Bcl-2, IL-6, TNF-α, and MMP-9, suggesting induction of apoptosis together with attenuation of inflammatory and metastatic pathways. Notably, these effects were achieved without perturbing cellular redox balance since the total antioxidant status/total oxidant status (TOS) ratio was preserved. These results underscore DZ-NS as a nanotechnology formulation that can boost the bioactivity of daidzein. Ahmed et al. [166] synthesized folic acid–conjugated daidzein solid lipid nanoparticles (DZN-FA SLNs) (223 nm particle size, 20.3% polydispersity index, −20 mV zeta potential) to improve delivery and anticancer activity against colon cancer cells. Caco-2 cells incubated for 48 h with DZN-FA SLNs presented significant growth inhibition, with an IC_50_ value of 10 µg/mL, significantly lower than that of free daidzein or unconjugated formulations. Mechanistically, the enhanced cytotoxicity was caused by increased cellular uptake through folate receptor-mediated endocytosis, in addition to the known activities of daidzein for apoptosis induction, tyrosine kinase inhibition, and modulation of PI3K/Akt and Wnt/β-catenin signaling. This system of nanoparticles also exhibited controlled drug release (53% over 48 h), high entrapment efficiency (~72%), and stability, attesting to its potential as a targeted cancer therapy for colon cancer.

#### 4.3.2. Lung Cancer

Oncu et al. [167] explored the anticancer activity of daidzein nanosuspension, which was developed to address the low-water solubility and bioavailability issues of daidzein in A549 cells. The cells were incubated for 24 h in the daidzein nanosuspension solution, and cytotoxicity was assessed using an MTT assay. A significant increase in antiproliferative activity was achieved for the nanosuspension, where IC_50_ was 25.23 µM, compared to unconjugated daidzein, where IC_50_ was 835 µM. The mechanistic results showed that the daidzein nanosuspension did not increase total oxidant status or oxidative stress index but significantly decreased these values compared to cisplatin treatment, suggesting that its cytotoxic effect does not involve induction of oxidative stress. Moreover, the nanosuspension was unable to induce the activation of caspase-3 at both the protein and mRNA levels, suggesting the absence of classical caspase-dependent apoptosis, while the expression of TGF-β1 remained largely unchanged. Notably, daidzein nanosuspension caused a substantial suppression in the gene expression of MMP-9. Overall, the above results indicate that the enhanced anticancer potential of daidzein nanosuspension arises due to the reduction in MMP-9 expression, without altering caspase-3 mediated apoptosis or inducing oxidative stress in lung cancer cells.

#### 4.3.3. Prostate Cancer

A very recent and innovative study by Xie et al. [56] has addressed the critical challenge of drug resistance in castration-resistant prostate cancer, which is driven by the overexpression of the androgen receptor (AR). The researchers discovered that daidzein itself possesses a high affinity for AR and can downregulate its levels. Leveraging this, they developed a novel, daidzein-based redox-responsive self-therapeutic nanocarrier to deliver enzalutamide (Enz), a standard AR antagonist. These nanoparticles (D44DA@Enz NPs) were designed to exploit the high redox state of the tumor microenvironment. Upon reaching the tumor site, the nanocarrier facilitated the degradation of AR via the ubiquitin-proteasome pathway, while simultaneously releasing Enz to inhibit AR nuclear translocation. This in vivo study demonstrated that this dual-targeted approach resulted in approximately 40-fold higher accumulation of the drug at the tumor site and exhibited exceptional antitumor activity with minimal systemic toxicity. This study not only confirms daidzein’s role as an AR degrader but also presents a highly promising self-therapeutic nanoplatform for overcoming drug resistance in advanced prostate cancer. However, current nanoformulation studies are predominantly limited to in vitro models, with minimal in vivo validation.

**Table 8:** In vitro and in vivo anticancer studies of daidzein-based nanoformulations.

Formulation Tested	Cell Line/Animal Model Used	Concentrations/IC_50_ and Duration	Anticancer Effects	Mechanisms	References
*Colon cancer*					
Chitosan-encapsulated daidzein formulation (in vitro)	HT-29	25–100 µg/mL, 48 h	┴Proliferation; ↑apoptosis	↑Caspase-3	[164]
Daidzein nanosuspension (in vitro)	Caco-2	35.3 μM, 24 h	↑Apoptosis	↑p53; ↓Bcl-2; ↓IL-6; ↓TNF-α; ↓MMP-9	[165]
Folic acid–conjugated daidzein solid lipid nanoparticles (in vitro)	Caco-2	10 µg/mL, 48 h	↑Cytotoxicity; ↑apoptosis	┴Tyrosine kinase	[166]
*Lung cancer*					
Daidzein nanosuspension (in vitro)	A549	25.2 μM, 24 h	↑Cytotoxicity	↓MMP-9	[167]
*Prostate cancer*					
Daidzein-based redox-responsive self-therapeutic nanocarrier (D44DA@Enz NPs) (in vivo)	22RV-1 tumor-bearing mice xenograft model	5 mg/kg (once every 3 days for a total of three doses; 21 d)	↓Tumor growth	↓AR degradation; ↓AR nuclear translocation; ↑accumulation in tumor	[56]

The arrows indicate the decrease (↓) or increase (↑) and inhibition (┴). Abbreviations: AR, androgen receptor; Bcl-2, B-cell lymphoma 2; IL-6, interleukin-6; MMP-9, matrix metalloproteinase-9; TNF-α, tumor necrosis factor-α.

## 5. Mechanistic Insights, Challenges, Limitations, and Translational Considerations

Daidzein, chemically an isoflavone and belonging to the phytoestrogenic family, has a significant medicinal importance for various pharmacological effects. The anticancer effects of daidzein have been investigated in a variety of different models. Overall, however, the evidence for daidzein’s anticancer properties is somewhat variable. Various studies have explored its effects in brain cancer, breast cancer, cervical cancer, colon cancer, choriocarcinoma, gastric cancer, hepatocellular cancer, lung cancer, oral squamous cell cancer, osteosarcoma, ovarian cancer, pancreatic cancer, prostate cancer, and skin cancer.

**Figure 4:**
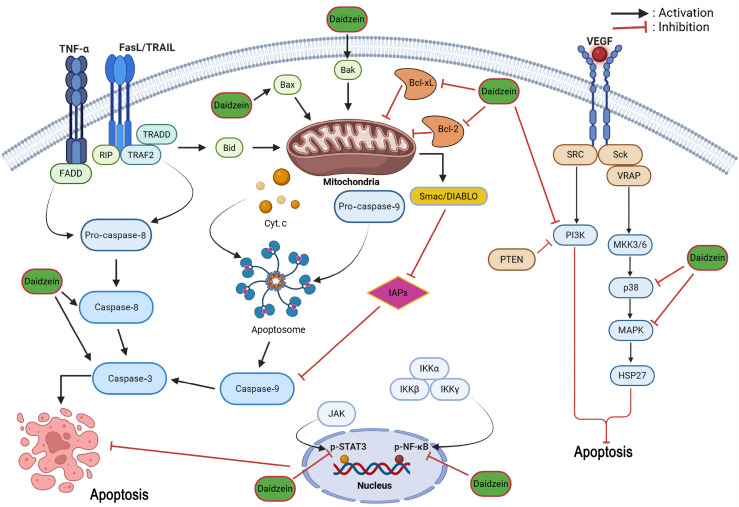
Overview of the antagonistic effect of daidzein in promoting apoptotic cell death in cancer cells. Major pathways involved in exhibiting such effects are TNF-α, NF-κB, VEGF, and FasL/TRAIL, causing intrinsic apoptotic pathway modulation. Abbreviations: Bak, Bcl-2 homologous killer; Bax, Bcl-2-associated X protein; Bcl-2, B-cell lymphoma 2; Bcl-xL, B-cell lymphoma-extra-large; Bid, BH3-interacting death domain agonist; DIABLO, direct inhibitor of apoptosis protein-binding protein with low pI; FADD, Fas-associated death domain; FasL, Fas ligand; HSP27, heat shock protein 27; IAP, inhibitor of apoptosis proteins; JAK, Janus kinase; MAPK, mitogen-activated protein kinase; MKK, mitogen-activated protein kinase; NF-κB, nuclear factor-κB; PI3K, phosphoinositide 3-kinase; PTEN, phosphatase and tensin homolog; RIP, receptor-interacting protein; Smac, second mitochondria-derived activator of caspase; STAT3, signal transducer and activator of transcription 3; TNF-α, tumor necrosis factor-α; TRAF2, tumor necrosis factor receptor-associated factor 2; TRADD, tumor necrosis factor receptor type 1-associated death domain protein; TRAIL, tumor necrosis factor-related apoptosis-inducing ligand; VEGF, vascular endothelial growth factor; VRAP, VEGF receptor-associated protein.

Daidzein has been reported to exhibit anticancer effects in vitro, as assessed against numerous forms of cultured cancer cell lines, in a controlled laboratory environment. Similar oncopreventive and therapeutic effects of daidzein have been inferred from preclinical in vivo studies also. The majority of the studies have signified apoptotic cell death in cancer cells by inflicting mitochondrial damage mediated through activation of caspase-3, caspase-9, and PARP, followed by dysregulation of the Bcl-2/Bax ratio in the cancer cells. Simultaneously, suppression of the PI3K-Akt pathway, aberrant formation of p-mTOR, and phosphorylation of FAK are the major contributory pathways that have been actively suppressed post-treatment with daidzein (Figure 4), which illustrates the apoptotic signaling pathways that are activated following the treatment with daidzein, including those related to mitochondrial function, caspase activation, Bcl-2 proteins, TNF-α/NF-κB, and FasL/TRAIL. Damage to DNA and various epigenetic modifications have also been well explained by daidzein application in various carcinomas. Formation of ROS and probiotic lysate was considered as the major cellular changes that caused degradation of carcinoma cells.

**Figure 5:**
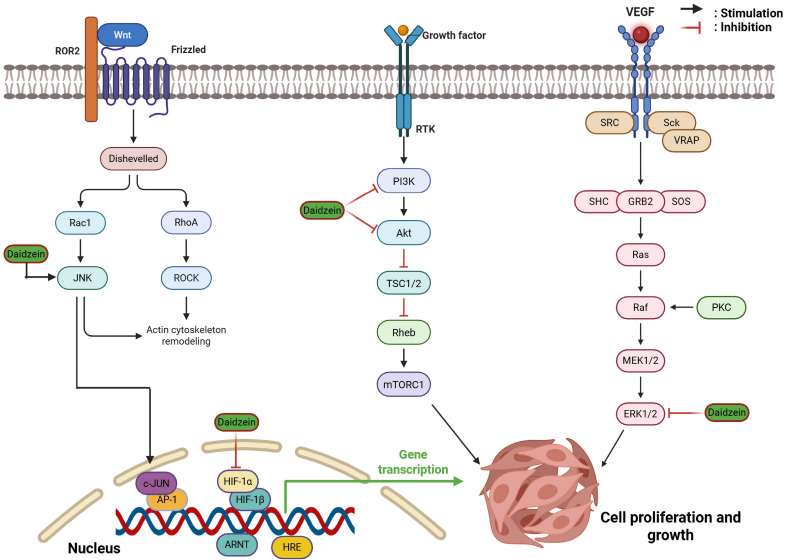
Role of daidzein in ameliorating cancer cell proliferation and growth following major mechanistic pathway alteration. Three major pathways, such as Wnt/β catenin, VEGF, and PI3K/Akt pathways, are the targeted ones due to the direct antineoplastic activity of daidzein. Abbreviations: Akt, Ak stain transforming; AP-1, activator protein-1; ARNT, aryl hydrocarbon receptor nuclear translocator; ERK, extracellular signal-regulated kinase; GRB2, growth factor receptor-bound protein 2; HIF-1, hypoxia-inducible factor-1; HRE, hypoxia response element; JNK, c-Jun N-terminal Kinase; mTORC1, mammalian target of rapamycin complex 1; PI3K, phosphoinositide 3-kinase; PKC, protein kinase C; ROCK, Rho-associated coiled-coil containing protein kinase; ROR2, receptor tyrosine kinase-like orphan receptor 2; SHC, Src homology and collagen adaptor protein; SOS, son of sevenless; TSC, tuberous sclerosis complex; VEGF, vascular endothelial growth factor, VRAP, VEGF receptor-associated protein; Wnt, wingless-related integration site.

Reduction in cell viability and proliferation serves as a prime mechanism adopted by daidzein to abolish cancerous cell growth. In many cancer cells, daidzein stimulated the formation of SOD and GSH, with significant retardation of TBARS formation. These changes were responsible for lowering the viability of cancer cells and for the quick destruction following programmed cell death. Cancer cell growth prevention by daidzein was also found due to modifications in lncRNA expression, as confirmed from a proteomics study. Additionally, Wnt/β catenin pathway, PI3K/Akt/mTOR pathway, and ERK1/2 mediated cell growth were significantly downregulated after treatment with daidzein (Figure 5), which summarizes the inhibition of several key signaling pathways, such as Wnt/β-catenin, PI3K/Akt, and VEGF signaling, which are all necessary for tumor growth and angiogenesis [168,169,170,171]. A small number of studies utilized molecular docking or molecular dynamics simulations to predict potential protein targets for the identified compounds, such as EGFR tyrosine kinase [72]. While these in silico techniques can identify potential targets and provide insights into the potential binding of small molecules to those targets, the docking scores do not indicate actual binding of that compound to the target protein. Thus, these results are merely preliminary results, and these techniques were not utilized in this review to draw conclusions regarding the activity of the natural products or their derivatives. More experimental validation of these potential targets is required prior to considering them as potentially active molecules regarding the described biological processes.

**Figure 6:**
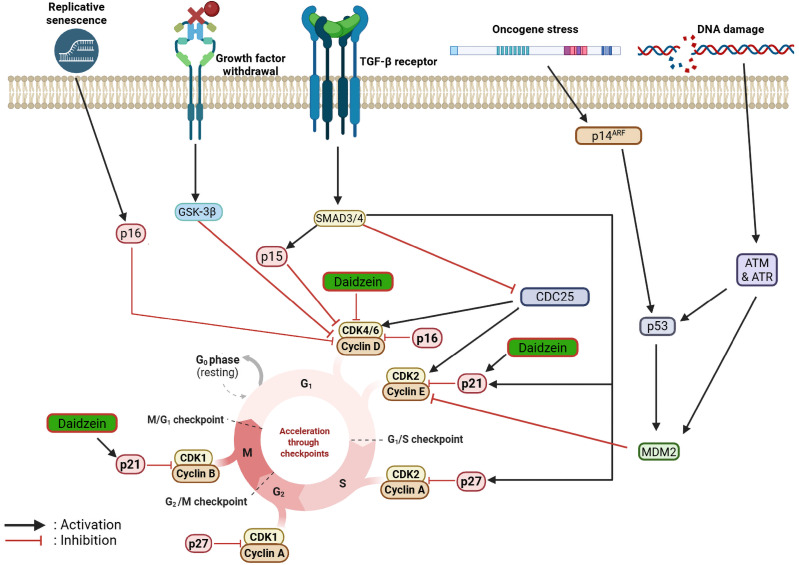
Effect of daidzein in cell cycle modulation due to direct effects of replicative senescence, growth factor withdrawal, stimulating TGF-β/SMAD3/4 pathway, oncogene stress, and inducing DNA damage. Abbreviations: ATM, ataxia-telangiectasia mutated; ATR, ataxia-telangiectasia and Rad3-related protein; CDC25, cell division cycle 25; CDK, cyclin-dependent kinase; GSK-3β, glycogen synthase kinase-3β; MDM2, mouse double minute 2; SMAD, suppressor of mothers against decapentaplegic; TGF-β, transforming growth factor-β.

The capacity of daidzein to trigger cell cycle arrest has been extensively validated across numerous forms of cancer cell lines. Modulation in CDKs expression and other checkpoint proteins for cell division arrested cell multiplication either in G_1_/S or G_2_/M phase (Figure 6), which depicts the regulatory network involving CDKs, checkpoint proteins, the DNA damage response, and the TGF-β/SMAD signaling pathway that contributes to the arrest of the cell cycle in the presence of DNA damage. Various factors, such as replicative senescence, DNA damage, withdrawal of growth factor, and activation of TGF-β receptors are the pinnacle modifications leading to cell cycle arrest [172]. However, several of these observations actually relate to downstream stress responses to the treatment with daidzein. In addition to the studies already reviewed, other studies that analyzed the effects of daidzein on cancer cells in vitro have also reported the observation of various other changes to the cells. For instance, many of these in vitro studies have indicated changes to Bax/Bcl-2 ratios, levels of caspases, levels of ROS within the cells, and changes to the membrane potential of the mitochondrial membrane of those cancer cells. Furthermore, while there have been studies that indicate changes to the levels of various signaling pathways, such as PI3K/Akt/mTOR, NF-κB, STAT3/FAK, Wnt/β-catenin, MAPK/ERK, and JNK, within the cancer cells following exposure to daidzein, these studies are generally correlational in nature. Thus, additional studies would be beneficial to determine which of these pathways are actually causally related to the anticancer activity of daidzein.

Combinatorial studies involving daidzein with another phytochemical or synthetic chemotherapeutic agent have also been proven to exhibit potent synergistic effects. As assessed against various forms of cancer, daidzein potentiated the activity of other natural compounds against cancer cells both in vitro and in vivo. Modulation of some major mechanistic pathways, such as minimization of STAT3 and FAK phosphorylation, led to mitochondrial damage and caused cell death following the apoptotic pathway. Downregulation of CDKs and cyclins halted cell cycle progression, leading to an anti-tumorigenic effect. This synergistic approach is very much crucial for a targeted antineoplastic effect, with aberrating the chances of developing chemoresistance.

Despite the plethora of in vitro and in vivo studies that demonstrate the anticancer activity of daidzein, there are some challenges to its clinical application. As a phytoestrogen, daidzein can bind to both estrogen receptor α (ERα) and ERβ. Studies on breast cancer have found that higher concentrations of daidzein led to decreased expression of ERα and increased expression of ERβ, both of which are associated with reduced proliferation of the cancer cells [71,72,119]. ERβ has been shown to have antiproliferative properties, whereas ERα has proliferative effects on breast cancer cells. However, at lower concentrations (≤1 μM), daidzein stimulates proliferation in ER-positive breast cancer cells (MCF-7) [66,68]. The estrogenic effects of daidzein are of concern for cancers that are sensitive to estrogen. In cells with low levels of endogenous estrogen (postmenopausal women, for example), daidzein may have a proliferative effect on these cells. At high levels of estrogen, however, daidzein may exhibit antagonistic properties toward estrogen. Furthermore, daidzein has been shown to upregulate the oncogenic transcription factor BRF2 in female mice but repress its expression in male mice [39]. Similar effects may be seen in prostate and ovarian cancer cells. Thus, these findings suggest that daidzein may have either a positive or negative effect on these cancers, depending upon the individual patient’s characteristics. Consequently, future clinical studies would need to account for these variables of each patient.

Several major concerns of using daidzein for pharmacological activities are its poor aqueous solubility, low bioavailability, rapid hepatic and intestinal metabolism through glucuronidation and sulfation reaction, followed by immediate systemic elimination. A significant challenge to the translation of these effects to humans is the difference between the concentrations that are effective in vitro versus those that can be achieved in human plasma. Table 3 summarizes the concentrations of daidzein and glycitein that are required to exhibit the effects described above, which generally range from 10 to 100 μM (with concentrations of >50 μM often required for cancer cell lines, such as glioma, liver, and osteosarcoma). However, plasma concentrations from human studies of women who consume soy diets or isoflavone supplements reach only 0.1 to 1 μM of daidzein; higher doses of isoflavones reach only 2 to 5 μM in plasma [173,174]. These lead to the hindrance of translating promising preclinical results into effective clinical manifestation. Furthermore, most in vitro studies utilize concentrations of daidzein that are much higher than those that are achievable in the blood of healthy individuals. Micromolar concentrations of daidzein have been detected in the blood of healthy individuals, but higher concentrations of daidzein have been utilized in experimental in vitro studies. These higher concentrations can lead to non-specific effects on the cells that are being studied, which may lead to an underestimation of the true anticancer properties of daidzein. Thus, these studies should be interpreted with caution, and future studies should utilize doses of daidzein that are more likely to be present in the blood of individuals with cancer.

The review also does not sufficiently highlight any contradictory findings within the literature regarding daidzein. For example, daidzein has been found to exhibit biphasic effects of soybean; low concentrations of daidzein stimulate the proliferation of cancer cells that are positive for ERs, while higher concentrations of daidzein inhibit the proliferation of those cancer cells or cause them to undergo apoptosis [66,68,87]. These effects are especially prevalent in cancers that are dependent upon hormones, such as breast, prostate, and ovarian cancers. Furthermore, studies show that the effect of daidzein may even be dependent upon the sex of the organism; daidzein has been shown to upregulate the oncogene BRF2 in female mice [39]. These contradictory effects of daidzein were not discussed within the original research study, and thus limit the ability of that publication to draw conclusions regarding the anticancer potential of daidzein. Future studies must discuss the dose-dependency and potential context-dependency of daidzein’s anticancer properties to avoid making generalizations of its effects from preclinical studies.

Furthermore, most of the studies used different models in their experiments. The in vitro experiments used a variety of cancer cell lines from both human and rodent sources, and used different concentrations of daidzein, for example, ranging from the submicromolar to several hundred micromolar concentrations, and used different lengths of treatment, from 24 to 120 h. The in vivo experiments used models ranging from daidzein administered to immunodeficient mice with xenograft tumors, chemically induced cancers (DMBA, DMH/DSS, DENA/CCl_4_, and benzo(a)pyrene), rodent strains, and sexes, and implantations of human or rodent cancers into the appropriate organs of those rodents. Thus, each of these different models and routes of administration introduces variability into each of these studies that prevents direct comparisons between their results. Accordingly, while the studies have classified their results according to the types of cancers that were studied, a direct comparison between any of those studies would be difficult to make due to these differences. Therefore, future studies can help to address these issues by employing standardized models of cancer, direct comparisons of daidzein effects within the same model systems, and studies that can evaluate the effects of these different models on the effects of daidzein.

Another factor that also requires consideration is the quality of the preclinical data that exists for daidzein. The studies that are performed describe daidzein’s ability to exhibit cytotoxicity within cancer cell lines as its “anticancer activity.” However, induction of cell death within transformed (cancer) cell lines does not necessarily indicate any selectivity for inducing death in cancer cells rather than normal cells, especially where the activity is thought to be due to the non-specific effects of the compound. None of the studies describes any attempts at measuring the cytotoxicity of daidzein in normal (non-tumorigenic) cell lines. While some of the studies reported that daidzein exhibited less toxicity in normal cells than cancer cells [73,75], there were no studies that performed rigorous assays to prove such selectivity for cancer cells. Overall, these results suggest that daidzein may exhibit general cytotoxicity, but not necessarily anticancer selectivity. Preclinical studies would be beneficial to perform that aim to determine daidzein’s toxicity for normal cells in order to more accurately evaluate its potential.

In the present review, discussion of signaling pathways is largely descriptive. While daidzein is often associated with the modulation of several oncogenic cascades (including NF-κB, PI3K/Akt/mTOR, MAPK/ERK, JNK, STAT3, FAK, and Wnt/β-catenin), most of the studies that have examined these pathways only report the change in the activity or expression levels of proteins downstream of these cascades (such as p-Akt, p65-NF-κB, and cyclin D1). Furthermore, most of these studies have not used methods that would suggest a specific targeting of these pathways (such as using gene knockdown methods, inhibitors that can rescue the effects of daidzein, or proteomic analyses to determine which proteins are targeted by daidzein). Thus, it is not currently possible to determine if daidzein has targeting effects for only one of these signaling pathways, or if it acts on multiple targets within the cancer cells. Consequently, future studies are required to investigate the mechanism of action of daidzein for these cancer cell targets. This limitation is consistent with the broader observation that many reported signaling alterations following daidzein treatment are correlative and require further causal validation.

The quality and limitations of the cited preclinical studies were not sufficiently evaluated or reported in many of the original research articles. Most in vitro studies used standard cytotoxicity assays (MTT and trypan blue) at high concentrations of daidzein (usually in the range of 10–200 μM), often exceeding plasma levels of daidzein in human adults (typically, 1–5 μM). Another major limitation of the literature discussing the anticancer properties of daidzein is its use of supraphysiological concentrations of daidzein in many of the studies. The concentrations of daidzein that are often utilized in in vitro and in vivo research studies are much higher than those that can be naturally present in the plasma of human beings. Furthermore, natural daidzein has limited solubility in aqueous solutions, poor bioavailability after being ingested orally, and is rapidly eliminated from the human body. Additionally, many of the beneficial effects of daidzein are thought to be due to its biotransformation into another compound called equol, which is dependent upon the presence of certain microbiota in the human digestive system; only around 30–50% of individuals have the microbiota necessary to naturally convert daidzein to equol. Thus, these pharmacokinetic limitations to daidzein’s bioavailability and bioactive form suggest a potential gap between the research studies and actual exposure to daidzein in human populations, indicating a need for caution in the interpretation of current study results and implications for future studies.

Furthermore, many in vitro studies did not include normal, non-tumorigenic control cells, making it impossible to determine if the effects of daidzein were specific to the cancer cell lines that were used. Studies also did not perform assays to determine if daidzein affected the activation of various signaling pathways (NF-κB, PI3K/Akt, STAT3, and MAPK), though these pathways are often correlated with cancer and are thus inherently a limitation of those studies.

**Figure 7:**
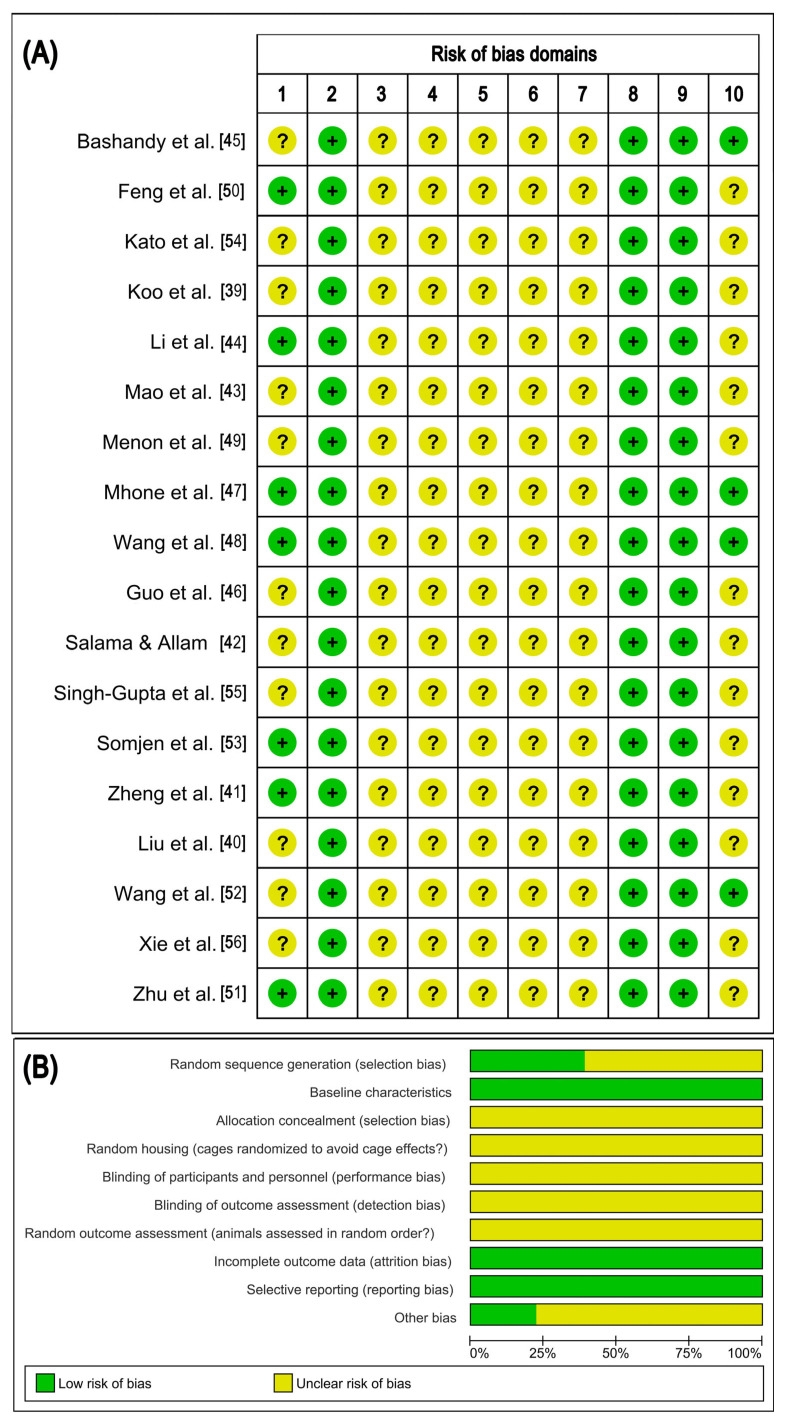
RoB assessment of in vivo studies on the anticancer activity of daidzein. The quality of each included animal experiment was assessed for potential biases using the SYRCLE RoB assessment tool. (**A**) RoB summary showing domain-wise judgments for each included study (green: low risk; yellow: unclear risk) [39,40,41,42,43,44,45,46,47,48,49,50,51,52,53,54,55,56]; (**B**) RoB graph illustrating the proportion of studies rated as low or unclear risk across the ten SYRCLE bias domains, highlighting frequent unclear risk judgments related to randomization and blinding procedures. The ten assessed domains included: (1) random sequence generation, (2) baseline group similarity or adjustment for confounders, (3) allocation concealment, (4) random housing of animals, (5) blinding of caregivers and/or investigators, (6) random outcome assessment, (7) blinding of outcome assessors, (8) appropriate handling of incomplete outcome data, (9) selective outcome reporting, and (10) other potential sources of bias. The analysis highlights frequent limitations in reporting related to randomization and blinding procedures across the included studies. The proportion of studies that are classified as having an “unclear risk” of bias is due to the insufficient reporting of methodological details rather than indicating a true risk of bias in the studies included in the review. Abbreviations: RoB, risk of bias; SYRCLE, Systematic Review Center for Laboratory Animal Experimentation.

The SYRCLE risk-of-bias assessment for the in vivo studies indicates that the majority of these studies had a moderate level of quality (Figure 7). Factors that contributed to the limitations of these studies may have included the small sample sizes for the studies, the lack of predefined endpoints for the studies, insufficient reporting of the number of animals used in each study, the various dosing regimens for daidzein, and the use of xenograft models of human tumors in immunodeficient mice. Furthermore, another potential limitation to the in vivo studies was the differences between laboratories, such as in the authentication of the cancer cell lines, the passage number of the cells used in each study, and the batch of daidzein that was used in each study. Publication bias toward positive results cannot be excluded, as negative or null findings are underrepresented. The restriction to using English-language publications may have introduced bias due to the potential for the omission of studies published in other languages but describing investigations into the effects of soy consumption on those who consume high amounts of soy within their diet. Thus, the study may have slightly limited comprehensiveness in its review of the effects of soy consumption.

The in vivo studies included in the review were not critically evaluated in any depth regarding parameters that relate to the relevance of the studies to humans, such as dosage, route of administration, and assessment of toxicity. The studies utilized doses between 5 and 300 mg/kg in animals, usually administered intraperitoneally or intravenously; higher doses than would be delivered to humans through oral administration of the compound. Human-equivalent doses were not utilized or discussed in most instances. Additionally, evaluations of the toxicity of the compounds to the animals were not performed in most cases. The experiments generally employed small number of animals (usually between 5 and 8), and did not explain the statistical power of the studies.

Moreover, evaluation of the RoB assessment tool developed by SYRCLE for the included in vivo research studying the anticancer activity of daidzein showed that the overall quality of methodology was moderate. Most of the included studies had a low risk of bias, including reporting bias, while major weaknesses were noted in the documentation of the fundamental aspects of the methodology, such as randomization, allocation concealment, masking of the treatment team, or masking of the outcome assessment. This rendered performance bias and detection bias sources of substantial uncertainty in the majority of the included experiments (Figure 7). This assessment was not used to weight or stratify the results of the synthesis of research studies due to the small number of research studies that were evaluated and the predominance of in vitro data within the reviewed literature. Quality assessments were not performed upon the in vitro research due to the fact that such studies comprise the majority of the research in this area. The limitations of in vitro research include the use of experimentally high concentrations of daidzein, the lack of studies that utilized non-tumorigenic cell lines, the lack of selectivity of daidzein relative to normal cells, the use of correlative analyses rather than causal analyses, and the different conditions under which the various in vitro research studies were performed. Each of these factors may indicate a potential overestimation of the effects of daidzein in the in vitro research, as well as reduce the trust that may be placed in the reproducibility of those studies. Future reviews of daidzein or research that intends to publish research on daidzein should employ more comprehensive quality assessment tools for in vitro research studies, as well as incorporate assessments of the risk of bias of in vitro research studies into their publications.

Despite the fact that most of the preclinical studies show that daidzein has potential anticancer properties, the shortcomings of these studies limit the generalizability of their findings. To further clarify the gap between the preclinical knowledge and the clinical applicability of that knowledge, Table 9 presents a summary of the main limitations of the preclinical research. Another major limitation of these studies is that they only used preclinical data. Since there are no clinical trials that have used daidzein for the treatment of cancer, it is impossible to conclude whether daidzein is effective in treating cancer. Additionally, the human studies that have been performed used mixtures of isoflavones from soy products, not daidzein alone. Thus, despite the fact that daidzein has shown anticancer properties in preclinical studies for various types of cancer, its clinical efficacy in humans remains uncertain and requires validation through well-designed clinical studies.

These limitations led to the development of daidzein-loaded nanoformulations for improved drug delivery and targeting with minimization of chemotherapeutic drug toxicity. However, despite the encouraging in vitro results for nanoformulations of daidzein, in vivo validation of such nanoformulations is lacking. Only one in vivo study was identified in the research on nanoformulations of daidzein. Thus, it is unclear as to whether these nanoformulations lead to improvements in the pharmacokinetic parameters of daidzein. Nanoformulations of daidzein are often proposed to increase the bioavailability of daidzein, as it is a poorly bioavailable phytochemical; however, there are no in vivo studies to back this proposition. Therefore, in vivo studies are needed to determine whether these nanoformulations of daidzein provide a therapeutic advantage over daidzein when employed in vivo.

As poor bioavailability of daidzein is a matter of concern, so novel approaches to encapsulate daidzein in various nano medications are some highlighted research topics in the modern era. Formulations such as microcapsules, solid lipid nanoparticles, and nanosuspensions have been explored as a means of enhancing the bioavailability and anticancer potential of daidzein; however, the benefits of these formulations have yet to be fully validated in vivo [175,176,177]. Additionally, the nanocarriers protect daidzein from degradation, thereby prolonging circulatory time and enhancing accumulation in tumor tissues for potent therapeutic efficacy. Other drugs, such as probiotics, which improve the gut microbiome for better biotransformation and bioavailability, can also be administered with daidzein for an overall better potency [74,178,179].

**Table 9:** Key translational gaps between preclinical findings and clinical applicability of daidzein.

Domain	Preclinical Evidence	Translational Limitation	Clinical Implication
Anticancer efficacy	Strong in vitro and in vivo tumor suppression across multiple cancer types	No clinical trials evaluating pure daidzein	Clinical efficacy remains unvalidated
Effective concentrations	Typically, 10–100 μM (sometimes higher)	Human plasma levels ~0.1–5 μM	Many observed effects may not be physiologically achievable
Mechanistic pathways	Modulation of PI3K/Akt, NF-κB, MAPK, Wnt/β-catenin, apoptosis pathways	Mostly correlative, limited causal validation	Uncertain primary mechanism of action
Selectivity	Cytotoxicity observed in cancer cell lines	Limited comparison with normal cells	Risk of non-specific toxicity
Hormonal effects	ERα/ERβ modulation, biphasic effects	Context-dependent (dose, sex, hormonal status)	Potential risk in hormone-sensitive cancers
Bioavailability	Improved in nanoformulations (in vitro)	Very limited in vivo validation	Uncertain pharmacokinetic advantage
Metabolism	Conversion to equol enhances activity	Only 30–50% individuals are equol producers	High inter-individual variability
In vivo models	Tumor inhibition in rodent models	Dosing, routes, and models are not clinically representative	Limited predictability for humans
Study quality	Moderate (SYRCLE RoB: unclear randomization/blinding)	Methodological variability	Reduced confidence in reproducibility

Additionally, it has been reported that daidzein can be converted by gut microbes into its more bioactive metabolite, equol. While equol exhibits enhanced biological activity compared to daidzein, the anticancer effects of daidzein are not entirely dependent upon equol production, evidenced by the studies that are published in the current review that show the anticancer effects of daidzein even in the absence of equol production by microbes in the gastrointestinal tract of those individuals who consumed daidzein-containing diets [180,181,182,183]. A study by Lv et al. [183] signified that only 30–50% of individuals have the microbiome necessary to convert isoflavones to equol, which may explain the differences in the responses of various individuals to these phytochemicals. Additionally, daidzein has high structural similarity with endogenous estrogen, which explains daidzein’s ability to bind with ERs [25]. This phenomenon can be devastating with respect to safety concerns in patients suffering from hormone-sensitive cancers, such as prostate and breast carcinomas, as daidzein can exert both agonistic and antagonistic effects, depending upon the hormonal environment [184,185,186,187,188].

## 6. Conclusions

Daidzein has demonstrated anticancer potential in a variety of different in vitro and in vivo models. Daidzein has been shown to impact various oncogenic pathways in these models, leading to its potential as a multi-targeted anticancer agent. The clinical relevance of these findings, however, remains to be established. Importantly, all of the evidence for the anticancer effects of daidzein has been derived from in vitro and preclinical in vivo studies. Consequently, no clinical trials investigating daidzein as an anticancer agent have been performed to date. Thus, any implications of these findings regarding the use of daidzein as an anticancer agent should be made with caution.

Despite the potential anticancer effects of daidzein, there are still a few challenges regarding its use as a cancer therapeutic agent. For instance, the concentrations of daidzein that are effective in most in vitro and in vivo cancer models have not yet been replicated in human plasma, limiting the potential bioavailability of daidzein. Furthermore, the estrogenic activity of daidzein presents challenges regarding its potential safety in cancers that are sensitive to estrogen levels. In order to effectively utilize daidzein as an anticancer agent, however, there are a few steps that can be taken. For instance, cancers that have demonstrated a robust response to daidzein in in vitro and in vivo models should be prioritized in clinical trials of daidzein. Such cancers include triple-negative breast cancer, glioblastoma, hepatocellular carcinoma, and castration-resistant prostate cancer. Additionally, any preclinical studies of daidzein should be performed according to higher standards of methodological rigor than those applied to most scientific studies to date (Figure 7) in order to mitigate the moderate risk of bias that is common in scientific research studies.

Furthermore, Phase I clinical trials of daidzein should be conducted to evaluate its safety and pharmacokinetics in humans. Following the current Phase Ib/IIa trials, similar studies may be performed with a focus on biomarkers, particularly in specific cancer types, and in consideration of the hormonal and metabolic status of the patients, and in the use of combination therapies with current treatments for the cancers. The trials will need to be carefully structured to evaluate treatment efficacy. Overall, therefore, daidzein appears to have notable promise as an anticancer agent; however, the ultimate success of daidzein in clinical use will depend upon overcoming the limitations of the current studies and incorporating the suggested future studies to fully realize its potential.

## Data Availability

Data sharing is not applicable to this article as no new data were created or analyzed in this study. This review synthesizes data from publicly available published studies, which can be accessed via their original sources.

## Abbreviations

ADA	adenosine deaminase
AHH	aryl hydrocarbon hydroxylase
Akt	Ak strain transforming
AO/EtBr	acridine orange/ethidium bromide
Apaf-1	apoptotic protease-activating factor 1
APC	adenomatous polyposis coli
APE/Ref1	apyrimidinic endonuclease 1/redox factor-1
AREG	amphiregulin
ASC	apoptosis-associated speck-like protein
ASK1	apoptosis signal-regulating kinase 1
Bax	B-cell lymphoma 2-associated X protein
Bcl-2	B-cell lymphoma 2
Bcl-xL	B-cell lymphoma-extra-large
BCRP	breast cancer resistance protein
Bid	BH3-interacting death domain agonist
BRF	butyrate response factor
CaCCs	calcium-activated chloride channels
CCl_4_	carbon tetrachloride
Cdc25C	cell division cycle 25C
CDK	cyclin-dependent kinase
CED	chitosan-encapsulated daidzein
c-FLIP	Fas-associated death domain protein like interleukin-1β-converting enzyme inhibitory protein
CI	combination index
c-Myc	myelocytomatosis oncogene
CRP	C-reactive protein
CXCL1	chemokine ligand
CYP	cytochrome P-450
cyt. c	cytochrome c
C115H-Dz	daidzein-conjugated methionine γ-lyase
DAPI	4′,6-diamidino-2-phenylindole
DENA	diethylnitrosamine
DMAB	3,2′-dimethyl-4-aminobiphenyl
DMBA	7,12-dimethylbenz[a]anthracene
DMH	1,2-dimethylhydrazine
DNA	deoxyribonucleic acid
DSS	dextran sulfate sodium
DZ-NS	daidzein nanosuspension
DZN-FA SLNs	folic acid–conjugated daidzein solid lipid nanoparticles
D4S	daidzein-4′-sulfate
EGFR-TK	epidermal growth factor receptor tyrosine kinase
EMT	epithelial–mesenchymal transition
ER	estrogen receptor
ErbB2	epidermal growth factor receptor 2
FADD	Fas-associated death domain protein
FAK	focal adhesion kinase
FANCC	Fanconi anemia complementation group C
FLICE	Fas-associated death domain protein like interleukin-1β-converting enzyme
FMK	z-VAD-fluoromethylketone
FOXO	Forkhead box O
FSP	ferroptosis suppressor protein
GGT	γ-glutamyl transferase
Gli1	glioma-associated oncogene homolog 1
GSH	reduced glutathione
GSK	glycogen synthase kinase
GSSG	glutathione disulfide
GTSE1	glutathione S transferase E1
Hh	Hedgehog
HIF	hypoxia-inducible factor
hTERT	human telomerase reverse transcriptase
IC_50_	half maximal inhibitory concentration
IFNγR	interferon-γ receptor
Ig	Immunoglobulin
IGF-1	insulin-like growth factor-1
IL	interleukin
JNK	c-Jun N-terminal kinase
KPNA2	karyopherin alpha 2
LDH	lactate dehydrogenase
lncRNAs	long non-coding RNAs
MAPK	mitogen-activated protein kinase
MCM2	minichromosome maintenance protein 2
MC1R	melanocortin 1 receptor
MDA	malondialdehyde
MKI67	murine Ki-67
MMP	matrix metalloproteinase
mRNA	messenger ribonucleic acid
MTT	3-[4,5-dimethylthiazol-2-yl]-2,5-diphenyl tetrazolium bromide
NF-κB	nuclear factor-κB
NGB	neuroglobin
Nrf2	nuclear factor erythroid 2-related factor 2
p-Akt	phosphorylated Ak strain transforming
PARP	poly (adenosine diphosphate-ribose) polymerase
p-ERK	phosphorylated extracellular signal-regulated kinase
PFT- α	pifithrin-α
PI	Hoechst–propidium iodide
PICO	Participants, Intervention, Comparator, and Outcomes
PI3K	phosphatidylinositol 3-kinase
p-mTOR	phosphorylated mammalian target of rapamycin
PPAR	peroxisome proliferator-activated receptor
PRISMA	Preferred Reporting Items for Systematic Reviews and Meta-Analyses
qPCR	quantitative polymerase chain reaction
RoB	risk of bias
ROPN1L	rhophilin-associated tail protein 1-like
ROS	reactive oxygen species
s.c.	subcutaneously
SOD	superoxide dismutase
STAT3	signal transducer and activator of transcription 3
STK4	serine/threonine-protein kinase 4
SYRCLE	Systematic Review Center for Laboratory Animal Experimentation
TBARS	thiobarbituric acid-reactive substances
TGF-β1	transforming growth factor-β1
TIMP	tissue inhibitors of metalloprotease
TMEM16A	transmembrane protein 16A
TNF-α	tumor necrosis factor-α
TOS	total oxidant status
TP53	tumor protein 53
TRAIL	tumor necrosis factor-related apoptosis-inducing ligand
TRAILR	tumor necrosis factor-related apoptosis-inducing ligand receptor
TRHR	thyrotropin-releasing hormone receptor
TRP	tyrosinase-related protein
TUNEL	terminal deoxynucleotidyl transferase dUTP nick-end labeling
uPAR	urokinase plasminogen activator receptor
VEGF	vascular endothelial growth factor
Wnt	wingless-related integration site
XIAP	X-linked inhibitor of apoptosis protein
XTT	2,3-bis-(2-methoxy-4-nitro-5-sulfophenyl)-2H-tetrazolium-5-carboxanilide
YAP-1	yes-associated protein-1
